# CalloseMeasurer: a novel software solution to measure callose deposition and recognise spreading callose patterns

**DOI:** 10.1186/1746-4811-8-49

**Published:** 2012-12-17

**Authors:** Ji Zhou, Thomas Spallek, Christine Faulkner, Silke Robatzek

**Affiliations:** 1 The Sainsbury Laboratory, Norwich Research Park, Norwich, NR4 7UH, UK; 2 Present address: RIKEN Yokohama Institute, Suehiro-cho, Tsurumi-ku, Yokohama City, Kanagawa, 230-0045, Japan; 3 Present address: Department of Biological and Medical Sciences, Oxford Brookes University, Oxford, OX3 0BP, UK

**Keywords:** Callose deposition, Quantification, Immunity, Flagellin, Flg22, Bacteria, Defence response, Oomycete, *Hyaloperonospora arabidopsidis*, Encasements, Pathogen, Image analysis

## Abstract

**Background:**

Quantification of callose deposits is a useful measure for the activities of plant immunity and pathogen growth by fluorescence imaging. For robust scoring of differences, this normally requires many technical and biological replicates and manual or automated quantification of the callose deposits. However, previously available software tools for quantifying callose deposits from bioimages were limited, making batch processing of callose image data problematic. In particular, it is challenging to perform large-scale analysis on images with high background noise and fused callose deposition signals.

**Results:**

We developed CalloseMeasurer, an easy-to-use application that quantifies callose deposition, a plant immune response triggered by potentially pathogenic microbes. Additionally, by tracking identified callose deposits between multiple images, the software can recognise patterns of how a given filamentous pathogen grows in plant leaves. The software has been evaluated with typical noisy experimental images and can be automatically executed without the need for user intervention. The automated analysis is achieved by using standard image analysis functions such as image enhancement, adaptive thresholding, and object segmentation, supplemented by several novel methods which filter background noise, split fused signals, perform edge-based detection, and construct networks and skeletons for extracting pathogen growth patterns. To efficiently batch process callose images, we implemented the algorithm in C/C++ within the Acapella™ framework. Using the tool we can robustly score significant differences between different plant genotypes when activating the immune response. We also provide examples for measuring the *in planta* hyphal growth of filamentous pathogens.

**Conclusions:**

CalloseMeasurer is a new software solution for batch-processing large image data sets to quantify callose deposition in plants. We demonstrate its high accuracy and usefulness for two applications: 1) the quantification of callose deposition in different genotypes as a measure for the activity of plant immunity; and 2) the quantification and detection of spreading networks of callose deposition triggered by filamentous pathogens as a measure for growing pathogen hyphae. The software is an easy-to-use protocol which is executed within the Acapella software system without requiring any additional libraries. The source code of the software is freely available at https://sourceforge.net/projects/bioimage/files/Callose.

## Background

Immunity against potentially infectious pathogens in plants involves a plethora of defence responses such as the deposition of callose, a 1–3 β-linked glucan polymer [1,2]. Imaging callose deposition has emerged as a widely used method to quantify the activity of plant defences to a range of different pathogens and pathogen-derived molecules (e.g. flg22 derived from bacterial flagellin) in different plant genotypes and mutants [3,4]. Measuring callose deposition is also a popular way to determine the activity of pathogen-derived virulence factors that interfere with the plant immune pathways to the benefit of the pathogen [5,6]. While the principle method of callose staining with aniline blue, followed by clearance of the leaves and taking microscopy images under UV light is well established [7], this approach is hampered by the fact that callose deposits can differ between replicate samples due to biological variation. Moreover, the pattern of spreading callose deposits can vary in response to different pathogen species as well as modes of infection [8,9]. To take these variations and differences into consideration, it is necessary to acquire a larger number of images, use more accurate solutions to quantify callose deposition and to measure pathogen growth patterns.

Improved quantification methods based on automated large-scale image processing will provide better measurements of defence responses, allowing the detection of subtle differences and thereby promoting our understanding of the mechanisms of plant immunity. The usefulness of quantitative bioimage analysis has been demonstrated for high-throughput microscopy in plant endomembrane trafficking [10,11] and monitoring plasmodesmata development [12]. Software solutions developed in these studies allowed comparative measurements of endosomal compartments and plasmodesmata revealing significant differences between different plant genotypes, chemical treatments, biotic and abiotic stresses and during plant development that in many cases were not possible to be observed by the human eye [10,12].

To date, measurements of callose deposits mostly rely on ImageJ [13] and FIJI [14] and/or some related plugins to extract quantifiable data from images of aniline-blue stained leaves [15,16]. Another emerging software package that contains similar functions for quantifying particle-like objects is ICY [17]. Although these software tools enable the detection of callose signals from microscope images, they are limited in their ability to accurately measure callose deposits. For example, we utilised both FIJI and ICY to process a typical callose image (Additional file 1). We followed the image processing workflow previously published and applied “Auto Threshold” and “Particle Analyze” functions in FIJI (Additional file 1A) and the “Spots Detector” method in ICY (Additional file 1B). The results suggest that the two software tools lacked sufficient functions to filter false detected objects as well as to reliably conduct shape/size measurements on detected callose deposits. The results were even more erroneous whilst batch processing callose images (e.g. using macro scripting in FIJI and selecting the “batch input detection” mode in ICY). Because most plant microscopy images contain autofluorescing noise signals derived from chloroplasts, xylem vessels, trichomes, and/or out of focus particle-like signals (typical background signals for plant leaf images), the current available image analysis tools can lead to incorrect detection and imprecise size/shape measurements. Furthermore, in practice these software tools are still semi-automated – manual inputs are required to enhance image quality, choose thresholding algorithms, and/or adjust filtering methods, which makes the image processing of callose deposition time consuming, error-prone, and not applicable for batch processing.

To overcome the above limitations, we developed CalloseMeasurer (v1.0) – a robust software solution that can automate the detection of callose deposits with a very high degree of accuracy and also recognise growth patterns of filamentous pathogen species. This software is based on the Acapella software framework (V2.0, PerkinElmer), which is designed for performing high content and high-throughput bioimage analysis. The usefulness and applicability of CalloseMeasurer are demonstrated with two example experiments.

## Results and discussion

### Algorithm

**Figure 1:**
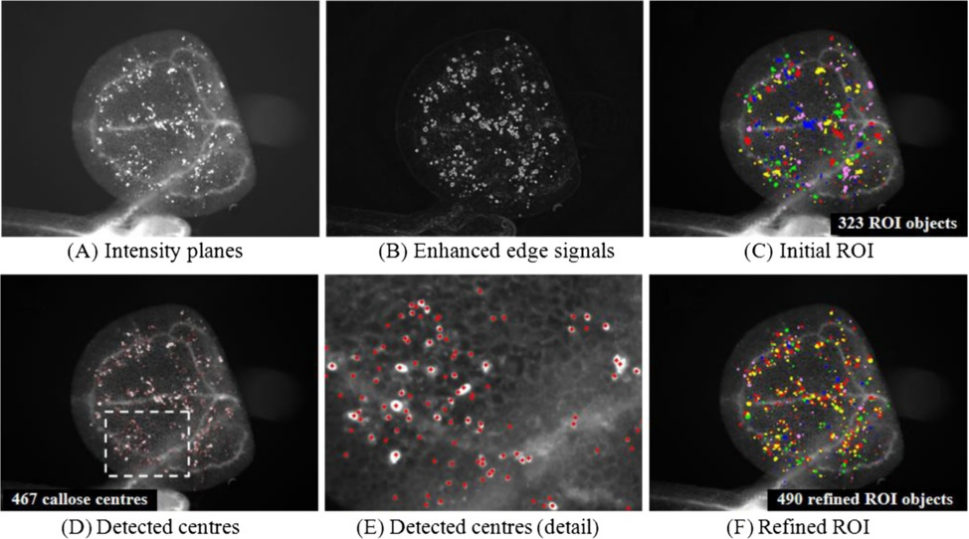
**The CalloseMeasurer analysis workflow for recognising ROI objects. **(**A**) The algorithm reads a series of callose image files into the software system, which are split into three planes – hue, saturation, and intensity value. Only intensity planes are used in the analysis. (**B**) As leaf vessel and mesophyll cell signals contain high intensity values, in order to differentiate them with callose deposition signals, images are transformed into their gradients so that callose edges can be highlighted. (**C**) Based on the processed images, image masks are applied to recognise ROI, which are randomly coloured. (**D**, **E**) Within the ROI, the algorithm detects centres of callose through finding local maxima of intensity. (**F**) ROI objects are rebuilt based on the detected callose centres.

**Figure 2:**
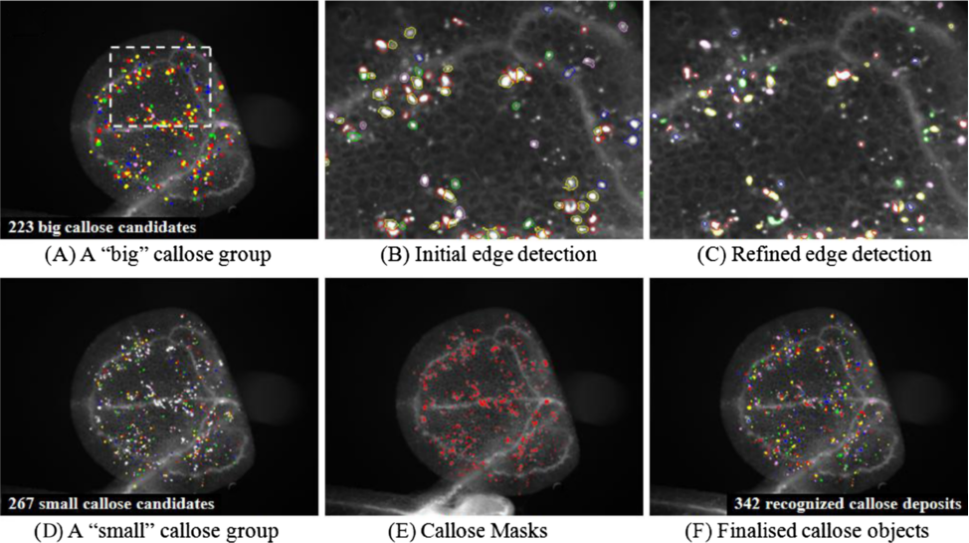
**The CalloseMeasurer analysis workflow for measuring callose deposits. **(**A**) The reconstructed ROI objects (Figure 2F) are divided into two groups – a “big” callose group (**A**) and a “small” callose group (**D**), both are randomly coloured. (**B**, **C**) In the “big” callose group, the border of callose is recalculated (callose objects are merged/separated based on the callose centres shown in Figure 2D). Some wrongly recognised callose candidates are removed. (**D**) Similar to the “big” callose group, the border of every small callose is measured and some wrongly detected ones are removed. (**E**, **F**) The refined “small” and “big” callose groups are merged (coloured red) and treated as callose deposition objects (randomly coloured). Quantifiable results (e.g., size, circularity, and intensity) are exported to two CSV files – one for every processed image and one for overall results.

**Figure 3:**
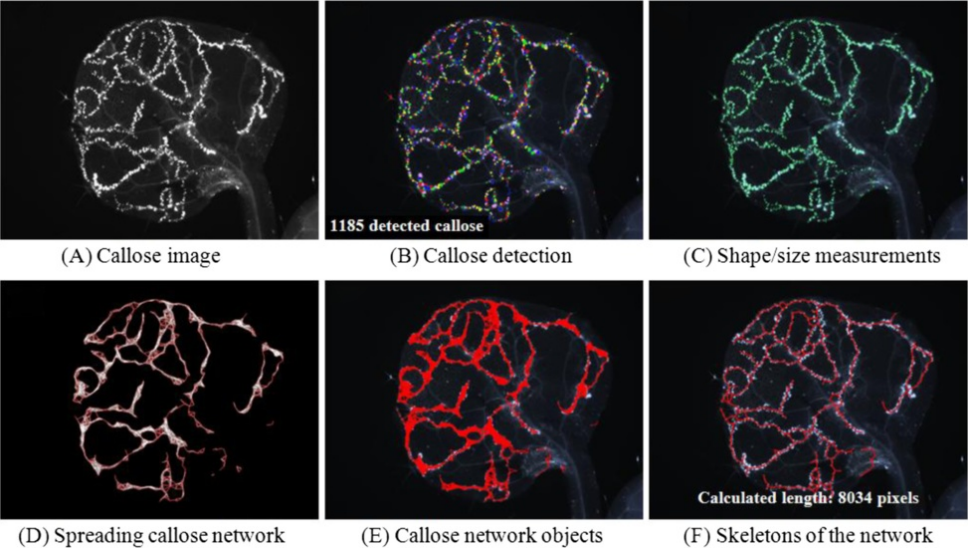
**The CalloseMeasurer analysis workflow for detecting callose spreading networks. **(**A**, **B**) If a user ticks the “Detect Callose Network” selection, a series of image files are read into the system and callose deposits (randomly coloured) will be detected by the algorithm. In this case, both small and big callose deposits are identified and used for constructing spreading callose networks. (**C**) Size, circularity, and intensity of the recognised callose deposits (coloured green) are measured. (**D**) A spreading callose network is constructed based on the recognised callose deposits. (**E**) Image masks are applied to the network (coloured red). (**F**) A filtering system is used to screen out unsuitable masks based on size, width, length, and shape. Skeletons are extracted from the refined callose network. Analysis results (e.g., the size and spreading length of callose deposits) are calculated and exported to the CSV file, which contains overall analysis results.

Similar to other image analysis approaches, we applied spot detection algorithms [18] to identify particle-like signals. To robustly filter background noise, we chose intensity analysis and adaptive thresholding methods [19] for removing fluorescent noise. To conduct sensitive edge-based measurements on size and shape, we implemented an edge detection method based on the border tracing approach [20]. Lastly, with the aim of recognising patterns of spreading callose deposits (i.e. filamentous pathogen growth patterns), we designed a tailored function to construct networks for spreading callose and extract skeletons of these networks, which was derived from the *network snakes* approach [21]. We integrated these methods into a powerful software solution that can filter out noise signals, split fused fluorescence signals, measure the size/shape of identified callose objects, and recognise patterns of spreading callose. In practice, we embedded the solution in a workflow for batch processing pathogen-induced callose images, which includes three main phases: detecting regions of interest (ROI) (Figure 1), measuring callose deposits (Figure 2), and recognising patterns of spreading callose deposits (Figure 3). Quantifiable results generated by CalloseMeasurer are saved in two CSV files (one containing results for every processed image and one for overall results).

### Implementation

We implemented the CalloseMeasurer algorithm in C/C++ together with a number of basic image analysis functions provided by the Acapella image library. In order to detect ROI, the algorithm reads callose images into the Acapella system and then divides them into three planes (e.g. hue, saturation, and intensity value planes). Only intensity planes are used during the image analysis (Figure 1A). As most of the input images contain high leaf vessel and mesophyll cell signals, the intensity planes are transformed into their gradients so that the border of callose signals can be highlighted (Figure 1B). Based on the processed images, a watershed method and image masks are applied to identify ROI (coloured randomly in Figure 1C), within which centres of every ROI object are located through the detection of local maxima of intensity (see Figure 1D and 1E). Centres with low intensity/contrast values are removed and remaining ones are treated as centres of callose deposit candidates. By taking into account raw image data (i.e. the intensity planes), recognised ROI objects, and callose deposition centres, the algorithm splits and rebuilds ROI (Figure 1F). The number of refined ROI objects could be different from the number of callose centres during the reconstruction of the objects list (see detailed algorithm implementation in Additional file 2).

As the size and shape of refined ROI objects are precisely measured, in order to perform sensitive measurements on callose deposits, the algorithm firstly divides the ROI objects into two size groups – a “big” callose group (Figure 2A) and a “small” callose group (Figure 2D), both are randomly coloured. For the “big” callose group, the border of every “big” object is recalculated based on their centres and some incorrectly separated (or merged) objects are remerged (or split). In the meantime, objects with low intensity/contrast or odd shapes are removed (Figure 2B and 2C). For the “small” callose group, a similar procedure is followed, which recalculates and filters small callose objects. After this step, refined “big” or “small” objects are merged (coloured red in Figure 2E) and treated as finalised callose deposits (randomly coloured in Figure 2F). Lastly, object measurements are conducted to quantify features such as size (in pixel^2^), perimeter (in pixel), circularity (according to the size and perimeter of recognised callose objects), and fluorescent signal intensity (between 0 and 255, which are extracted from the original intensity planes). Results are exported to two CSV files during the batch processing – one containing results for every processed image (including processed image name, callose index, size, circularity, and signal intensity) and one for overall results (including processed image name, callose number per image, average size and signal intensity of detected callose deposits). We included some examples of exported CSV files in Additional file 3.

When implementing the algorithm, we followed best practices in image processing and treated bioimages as data, so that feature selection could be mainly performed based on statistical analysis of features of image data sets [22]. As software filters (e.g. convolution and median filters) degrade biological image data, therefore we only extracted quantifiable results (e.g. intensity/contrast) from raw image data. In order to differentiate high background signals from callose deposits, we utilised global values at the image level (for processing images) and adaptive local values at the object level (for analysing objects). Moreover, we also applied some software engineering concepts (e.g. reusability and modularity) to the development of the software for improving software usability and functionality.

### Examples of applications

#### Measuring callose deposition in Arabidopsis thaliana triggered by bacterial flagellin

**Figure 4:**
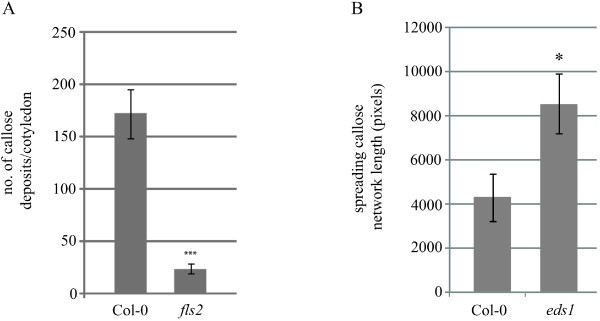
**Measurement of callose deposition following flg22 treatment and after. *Hpa *infection using CalloseMeasurer. **(**A**) The number of callose deposits following flg22 treatment was significantly greater on Arabidopsis Col-0 cotyledons than on cotyledons of *fls2 *mutant plants. (**B**) The spreading network of callose on *Hpa *infected leaves was significantly longer on *eds1 *mutant leaves than on Arabidopsis Col-0 leaves. Asterisks indicate p-values < 0.05 (*) and < 0.001 (***). Error bars represent the standard error.

The flg22 peptide derived from bacterial flagellin is a powerful agent to trigger callose deposits in plants [23]. We treated Arabidopsis cotyledons for 24 hrs with 1 μM flg22 and imaged fluorescence of aniline blue stained callose deposits [24]. To illustrate the high degree of accuracy of quantifying callose deposits using CalloseMeasurer we compared the flg22 responses from wild type plants and mutants in *flagellin sensing 2* (*fls2*), which encodes the receptor for flg22 and thereby causes insensitivity to this microbe-derived molecule [25]. The algorithm was able to detect 0–473 callose deposits per image sample and measure significant differences (student’s t-test) between these genotypes following flg22 treatment (Figure 4). We included some callose detection results (for both controlled and flg22-induced images) in Additional file 4 (Figure 1A and 2A).

#### Measuring callose deposition in Arabidopsis thaliana infected with an oomycete pathogen

Another strong infection stress that induces the accumulation of callose is the encasement of the haustoria of filamentous pathogens such as the oomycete *Hyaloperonospora arabidopsidis* (*Hpa*) on *Arabidopsis thaliana*[8]. For infecting plants, we spore inoculated seedling leaves with the *Hpa* strain Waco9 as previously described [26] and imaged leaves 6 days post infection. In order to detect the accumulated callose structure, we included a unique function in CalloseMeasurer, detecting “Callose Network”, to measure the spreading length of callose deposits. This functions as a biological measure of the total length of the hyphae that has undergone encasement. If the “Callose Network” option is ticked by users, the algorithm will process input images as previously described – detecting and measuring callose deposits from microscope images (Figure 3A-3C). After that, centres of every detected callose deposit are located and relevant centres are connected if the distance between two centres is shorter or equal to a defined distance threshold. In general, users can enter a specific value (in pixels) as the distance threshold. If no value has been entered, CalloseMeasurer will calculate a default distance value, based on which the spreading callose network will be constructed. The formula that calculates the default distance value can be seen as follows:

(1) $$\text{Distance}=\frac{1}{n}\overset{n}{\underset{i=1}{\sum }}D_{i}+\frac{1}{m}\overset{m}{\underset{j=1}{\sum }}d_{j}+\frac{1}{l}\overset{l}{\underset{k=1}{\sum }}F_{k}$$

(D_*i*_ is the diameter of the i^th^ object of the “big” callose objects list and n is the size of the list; d_*j*_ is the diameter of the j^th^ object of the “small” callose objects list and m is the size of the list; F_*k*_ is the full length of the k^th^ object of the detected callose objects list and l is the size of the list).

After connecting relevant callose centres, a spreading callose network is created (Figure 3D). Image masks can be applied to the network and CalloseMeasurer will discard masks with unsuitable size, width, length, and shape (Figure 3E). Remaining masks are saved in one objects list so that skeletons of these objects can be extracted (Figure 3F). The software measures the spreading length of callose deposits based on the extracted skeletons. The formula to perform the calculation is:

(2) $$\text{Length}=\overset{n}{\underset{i=1}{\sum }}\left(\frac{p_{i}-w_{i}}{2}\right)$$

(p_*i*_ is the measured perimeter of the i^th^ object of the callose skeleton objects list, w_*i*_ is the measured width of the callose skeleton objects, and n is the size of the callose skeleton objects list).

We included a variety of results of spreading callose networks in Additional file 4 (Figure 3A, based on good, fair and bad quality callose images). Examples of the quantification of spreading callose networks (e.g. the size and spreading length of the constructed networks) are included in Additional file 4. Furthermore, we quantified the length of hyphae with encased haustoria for Arabidopsis Col-0 wild type and the mutant *enhanced disease susceptibility 1* (*eds1*) (Figure 4). Mutant *eds1* plants are more susceptible to *Hpa* Waco9 when spore counts are measured [27]. Similar results were shown by CalloseMeasurer which detected significantly longer lengths of encased haustoria, indicating a more advanced infection. We detailed comments and the implementation of this novel function in Additional file 2.

### Limitations

Automated detection of callose deposition promises to be useful in many plant-pathogen interaction studies. It relies on a mixture of image analysis tasks such as image enhancement, signal filtering, object segmentation, object measurements, and feature selection. CalloseMeasurer offers the functionality for performing these tasks and allows researchers to robustly and accurately detect callose deposits for monitoring activities of plant immunity. Although the software framework (the Acapella software framework) used in the implementation is a commercial platform, it has been regularly used in bioimage analysis in many research areas [11,28,29]. Moreover, with the aim of sharing the software solution with the research community, we only chose basic image analysis modules/functions from the Acapella library during the development of the software. All functions used have counterparts in open-source bioimage analysis libraries such as ImgLib [30], which means that if necessary, the source code can be easily translated into an open-source software package with limited development efforts. In order to help users or developers to gain an in-depth understanding of CalloseMeasurer, we provide detailed user manual, detailed comments, and some experiment results in the Additional files. For testing purpose, users can obtain a free one-month trial version of the Acapella system from PerkinElmer.

## Conclusions

Along with the increasing importance of bioimage informatics in recent years, many bioinformaticians and computational biologists are dedicating themselves to developing novel computational techniques to extract meaningful data from large-scale biological image data [31,32]. A number of computational techniques such as signal processing, machine learning, data mining, mathematical modelling, and multi-dimensional data visualisation have been utilised to solve various data-intensive biological problems [33]. Following this trend, we previously developed algorithms for quantification of plant cells, plasmodesmata and endomembrane compartments and have now designed and implemented a novel software solution to measure the activity of plant immunity at the tissue level. In this study, we implement CalloseMeasurer, a new software solution for accurate quantification of callose deposits from large sets of bioimages. The unique features of CalloseMeasurer are: batch-processing of images; robust filtering of background noise signals common to plant fluorescent microscopy images; detection and measurement of callose deposits with high sensitivity and accuracy; and detection of spreading networks of callose. For demonstrating the usefulness of CalloseMeasurer, we presented two example applications that show quantitative differences in callose deposition between genotypes and detection of pathogen growth *in planta*.

## Methods

### Plant growth conditions

*Arabidopsis thaliana* plants were grown on Jiffy pellets (Jiffy Products International AS, Norway), or for sterile conditions on Murashige and Skoog (MS) medium [34] (Duchefa, Netherlands, order number M0256) under 10 hours or 16 hours of light at 20–22°C and 65% humidity.

### Bioassay for callose deposition and pathogen inoculation

For callose induction, flg22 was applied at 1 μM for 24 hrs, and callose deposits were stained with aniline blue and visualized as described before [27]. Briefly, ten-day-old seedlings were transferred to liquid MS media with and without 1 μM flg22, destained after 24 hours in acetic acid - ethanol (1:3) for four hours, washed twice with ddH_2_O, and incubated o/n in aniline blue solution (150 mM KH_2_PO_4_, 0.01% (w/v) aniline blue, pH 9.5). For infecting *A. thaliana* with the *Hpa* oomycete, suspensions of 5 × 10^4^ spores/ml of *Hpa* strain Waco were spray-inoculated onto 14-day-old seedling and incubated at high humidity at 18°C as previously described [26]. Infected leaves were stained for callose 6 days post-inoculation.

### Microscopy and image acquisition

Stained callose was visualized using an ultraviolet epifluorescence microscope (Zeiss Axiophot, Carl Zeiss AG, Oberkochen, Germany). All microscope images were saved in TIFF or PNG format.

### Image processing

**Figure 5:**
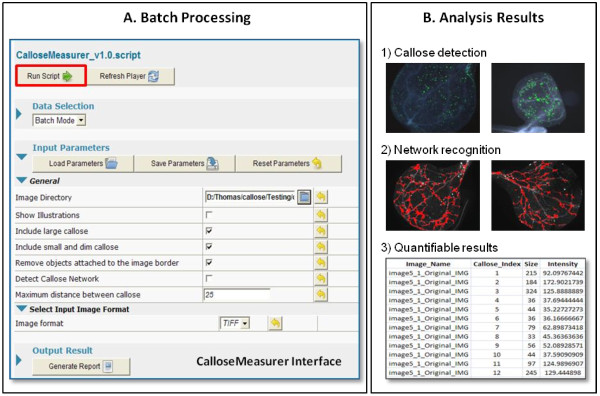
**A generic workflow of batch processing flg22-induced callose deposits image sequence using CalloseMeasurer. **(**A**) A series of callose images are batch processed by CalloseMeasurer (v1.0). (**B**) CalloseMeasurer detects callose deposits (highlighted in green) and identifies spreading callose networks (optional, coloured red). Generated images are saved as PNG files. Quantifiable callose features such as size, shape, fluorescence signal intensity, and area/length of spreading callose networks are exported to two CSV files for further statistical analysis.

After collecting callose images, we batch process images using CalloseMeasurer. Users are required to drag and drop the CalloseMeasurer script into the Acapella interface and then tick selection boxes according to their analysis requirements (e.g., image directory, image format, types of callose to measure, whether or not to construct spreading callose networks). After setting the input parameters, the analysis workflow can be initiated by clicking the “Run Script” button (Figure 5A). Following the batch processing, a set of PNG images with recognised callose deposits (coloured green) are generated. Features such as size, shape, and fluorescence signal intensity are measured based on every recognised callose deposit and saved in CSV files. If the “Detect Callose Network” option is selected, spreading callose networks are constructed (Figure 5B).

## Competing interests

The authors declare no competing interests.

## Authors’ contributions

SR, TS, and CF designed the research project. JZ created the image analysis algorithm and implemented the software. TS and CF performed the research and the image acquisition. JZ drafted the manuscript with help from SR and CF All authors read and approved the final manuscript.

## Supplementary Material

Additional file 1 Detecting callose deposition using FIJI and ICY. Click here for file

Additional file 2 CalloseMeasurer.script. Click here for file

Additional file 3 Examples of CSV files generated for results. Click here for file

Additional file 4 CalloseMeasurer analysis results for batch processing images and for constructing spreading callose networks. Click here for file
