# Supplementary material for: CalloseMeasurer: a novel software solution to measure callose deposition and recognise spreading callose patterns
Source: Plant Methods. 2012 Dec 17;8:49. doi: 10.1186/1746-4811-8-49 (PMC3571893; doi:10.1186/1746-4811-8-49)
Supplement: Additional file 1 — Detecting callose deposition using FIJI and ICY. [file 1746-4811-8-49-S1.pdf]

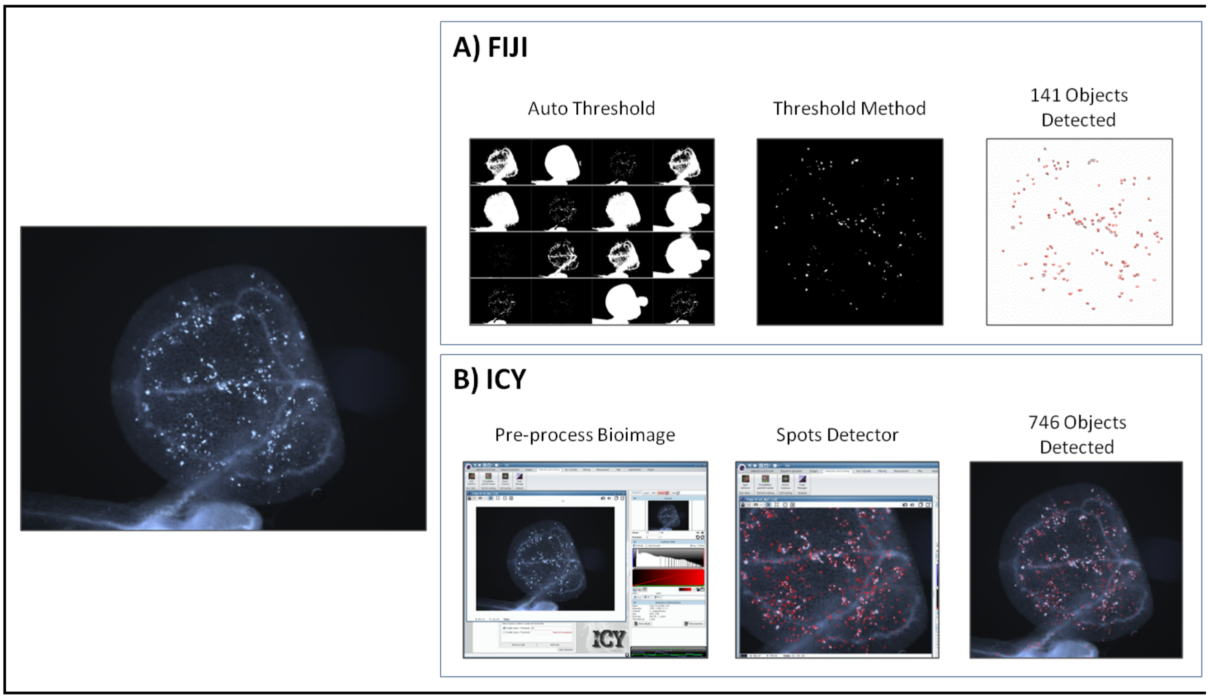

Figure 1 Detecting callose deposition using FIJI and ICY

(A) Detecting callose deposits using FIJI – functions such as "Auto Threshold", "Make Binary", and "Analyze Particles" were applied during the image processing.

(B) Detecting callose deposits using ICY – "Spots Detector" module was used.
