# Supplementary material for: CalloseMeasurer: a novel software solution to measure callose deposition and recognise spreading callose patterns
Source: Plant Methods. 2012 Dec 17;8:49. doi: 10.1186/1746-4811-8-49 (PMC3571893; doi:10.1186/1746-4811-8-49)
Supplement: Additional file 2 — CalloseMeasurer.script. [file 1746-4811-8-49-S2.pdf]

```
//**************************************************************************************************************
//		Script name: CalloseMeasurer.script
//		Version: 1.0 - polished for publication
//
//
//		The Silke Robatzek Lab
//		The Sainsbury Laboratory, Norwich Research Park, Norwich, UK
//
//		Dr Ji Zhou
//		email: ji.zhou@tsl.ac.uk
//		Tel: + 44 (0) 1603 450316 (2316 internal)
//
//
//		System Requirements:
//				To allow use of this script, the Acapella framework (v2.0 or above) and Windows XP are required.
//
//
//		 User Manual:
//				This software solution is designed to analyse spreading callose deposits on a plant leaf tissue
//				and, if necessary, to construct spreading callose networks. Output fields (in a variety of CSV files)
//				can be used to quantify features such as size, shape, fluorescent signal intensity, and pathogen growth
//				patterns (e.g. total length and spreading area). In order to measure callose deposits as well as detect
//				callose spreading networks, users need to follow the following instructions:
//
//				*		Selecting "Image Directory" selection to tell CalloseMeasurer where to read microscopic images
//
//				*		If users would like to only detect only big callose deposits, please tick the "Include large callose" selection
//
//				*		If users would like to only detect only small callose deposits, please tick the "Include small callose" selection
//
//				*		If users would like to only detect both small and big callose deposits, please tick both "Include small callose"
//						and "Include large callose" selections
//
//				*		If users would like to preview analysed images within the Acapella, please tick "Show Illustration"
//						selection, otherwise analysed images will be exported to the result folder, which will be generated by
//						CalloseMeasurer during the batch process
//
//				*		If users would like to remove detected objects attached to the image border, please tick the "Remove objects
//						attached to the image border" selection.
//
//				*		If users would like to detect spreading callose networks, please tick the "Detect Callose Network" selection -
//						be aware that this function is time-consuming. Although computation time depends on computer hardware,
//						the version of the Acapella system used for the analysis, and the complexity of the pathogen growth patterns,
//						CalloseMeasurer normally needs to spend 5-6 minutes to construct a modestly complicated callose network.
//						However users could always enter a smaller distance threshold (in pixel value) before constructing callose
//						networks. The threshold can be entered in the input box "The Distance Threshold of Callose Networks".
//						The default value is 25 pixels, which can be changed by the software according to the callose diameters
//						and full length of recognised objects of callose deposits.
//
```

```
//			*	Users can select the input image format on the "Image Format" dropdown list - TIFF, PNG, BMP, and JPG
//				formats can be read and processed by CalloseMeasurer. The default input image format is TIF/TIFF files
//
//			This software solution performs image analysis functions such as image enhancement, object segmentation, filtering
//			noise objects, splitting fused callose signals, edge-based measurement, and construct networks for detecting pathogen
//			growth patterns in leaf tissues. New functions can be added as external procedures or internal functions.
//
//
//
//		Copyright:   (C) The Sainsbury Laboratory
//
//
//*************************************************************************************************************



///// Establish input parameters
set( formats= " \"bmp\", \"jpg\", \"png\", \"tiff\" " )

//*** INPUT PARAMETERS ***//
Input(filepath, NO, type="p", prompt="Image Directory: ", description="Please select the directory of input callose images...")
input(ShowIllustrations, NO, "Show Illustrations", "y", "YES- Output illustrations are depicted. No- Output illustrations are not shown.")
input(LargeCallose, Yes, "Include large callose", "y", "YES- Analysis will retain large callose. No- Objects will remove large callose objects during the detection.")
input(SmallCallose, Yes, "Include Small Callose", "y", "YES- Analysis will retain small callose (callose with low contrast will be removed as required). No- Objects will remove small and dim callose objects during the analysis.")
input(RemoveBorderObj, Yes, "Remove Objects Attached to The Image Border", "y", "YES- Analysis will remove objects attached to the image border. No- Objects attached to the image border will be retained.")
input(Callose_Network_Trigger, NO, "Detect Spreading Callose Networks", "y", "YES- Export an image that contains spreading callose network together with quantifiable attributes such as area and full length. No- No callose networks will be constructed.")
input(Network_Threshold, 25,"The Distance Threshold of Callose Networks","i","Enter the maximum distance in pixels between two callose objects when establishing callose networks - default value (25 pixels) might be changed according to the recognised callose objects")
input(IN_image_format, "TIFF" ,"Image format: Select Input Image Format", "s", "Possible values: \"PNG\", \"TIFF\",\"JPG\", \"BMP\". Format \"tiff\" is optimal for analysis and \"png\" images will be generated as processed images.")
//*** End of Input Parameters Section ***//


//*** READ IMAGES, PREPARE FILE SYSTEM FOR OUTPUT CSV FILES ***//
//*** READ in images into a vector ***//
set( imageformat = IN_image_format )
// Find the format of input images
If(at("p", IN_image_format, 1))
		Glob(filepath & "/*.*p*", ignorecase=yes)
		// Only compressed png or jpg or bmp files will be read into the Acapella system
End()
If(at("t", IN_image_format, 1))
		Glob(filepath & "/*.*tif*", ignorecase=yes)
		// Only tif/tiff files will be read into the Acapella system
End()

// Display a warning message in case no image can be found in the directory
```

```
If(files.size == 0)
	Warning("No image has been found - please check the image directory as well as the selected image format!")
	// Finish analysis as no image has been loaded
Else()
	If(!SmallCallose && !LargeCallose)
		Warning("At least one type of callose deposits should be selected - please check your selection for detecting callose deposits!")
		// Finish analysis as no image has been loaded
	Else()
		//*** Prepare a Result folder for saving CSV results and processed images
		set(IMG_Name = " ")
		Set(current_Date = __date__)

		//*** Prepare output Result folder
		set( namelength = length( files[0] ) )
		set( pathname = substr( files[0], 1, at("/", files[0], -1)))

		Set(CreateDir = pathname & current_Date & "_Results")
		Set(Output_File = current_Date & "_Overall_Results")
		Delete(FileInfo)
		FileInfo(CreateDir)

		If(!fileinfo.exists) // Make a directory for CSV files
			MkDir(CreateDir, 555) // Set the directory attributes - only required in case we run it on Linux machine
		End()

		//*** Prepare overall output CSV file - average callose size, intensity, spreading network
		printfopen(CreateDir & "/" & Output_File & ".csv")
		Printf("Image_Name#")
		Printf("Callose_Number#")
		Printf("Average_Callose_Size#")
		Printf("Average_Callose_Intensity#")
		If(Callose_Network_Trigger) // If callose network is required
			set(Network_Length = 0)
			Printf("Calculated_Spreading_Length#")
		End()
		Printf("Total_Spreading_Callose_area#\n")
		printfopen()
		//** End of preparing the CSV file for saving overall output results

		//*** Start to process input images in the vector ***//
		foreach(0..(files.size-1), file_counter)

			Printf("Start analysing image %s \n", files[file_counter])

			//*** Start to prepare CSV files for every processed image ***//
			Delete(IMG_Name)
			// Start to produce the output csv
			set(IMG_Name = substr( files[file_counter], at("/", files[file_counter], -1) + 1))
			Set(Experiment_Name = substr(IMG_Name, -1, (at(".", IMG_Name, -1) + 1)))
```

```
                    // Prepare output csv for every callose
                    printfopen(CreateDir & "/" & Experiment_Name & "_Callose_Results"& ".csv")
                    Printf("Image_Name#")
                    Printf("Callose_Index#")
                    Printf("Size#")
                    Printf("Circularity#")
                    Printf("Intensity#\n")
                    printfopen()
                    //** Finish preparing the output CSV file

////**** Step Zero: pre-process images ****////
                    Delete(Source_Path)
                    set(Source_Path = files[file_counter]) // one channel image
                    ReadImage(Source_Path) // Read image into the buffer
                    set(Callose_IMG = image)
                    HsvSplit(Callose_IMG)
                    Set(Callose_Value = value) // Only use value planes during the image analysis


          //// ANALYSIS STARTS FROM THIS LINE ONWARDS ////
          ///*** STEP ONE: Detect ROI ***///
           ///*** Step 1.1: Find ROI using  Edge Detection (Gradient) and Convolution ***///
                    //Transforms the image into its gradient
                    Gradient(image=Callose_Value, sobel=yes, directional= 0)
                    Set(Gradient_IMG = image)
                    expand(5, 0.25, image=Gradient_IMG) // Enhance the processed image
                    Set(Processed_IMG = image) // Process the image with Gradient
                    set(convolutionkernel=toimage(vec(1,4,16,4,1, 6,24,36,24,6, 1,4,16,4,1),5,3).image) // build up a convolution kernel
                    set(convolutionkernelfactor=convolutionkernel.sum)
                    convolution(image = Processed_IMG)
                    Set(Convolution_IMG = image) // Process images with a convolution filter, so that the sharp edge can be soften

                    // Use bright mask to find the central part of callose objects
                    Bright_Mask(Callose_Value, 5)
                    // using adaptive thresholding
                    ThresholdXX(TuneTH=1.25, Image=result)
                    Mask(Threshold=Threshold * 3.75, Image=result)
                    Mask2Stencil()
                    Stencil2Objects()
                    FillObjects()
                    CalcArea()
                    ObjectFilter(area > 5 && area < 2500)
                    CalcIntensity(Image=Callose_Value, Total= yes)
                    CalcAttr(AVG_Intensity, (intensity * 255)/area)
                    Set(Intensity_Threshold = objects.AVG_Intensity.mean)
                    object_contrast_general(reference = Callose_Value, ContrastDef = "WithoutCyto")
                    ObjectFilter(contrast > 0.25 || AVG_Intensity > Intensity_Threshold * 1.25)
                    set(ROI_Initial_BM=Objects)
```

```
        // Initial ROI objects have been detected

        // Start to detect mask based on Gradient image
        // using adaptive thresholding
        If(Convolution_IMG.mean / Convolution_IMG.median > 3.25) // image contains big intensity difference
                ThresholdXX(TuneTH=1.25, Image=Convolution_IMG)
                Mask(Threshold=Threshold * 6.5, Image=Convolution_IMG)
        Else() // image has relatively equally spreading intensity values
                ThresholdXX(TuneTH=1.25, Image=Convolution_IMG)
                Mask(Threshold=Threshold * 6.25, Image=Convolution_IMG)
        End()

        // Start to build up intial ROI objects list
        Mask2Stencil()
        Stencil2Objects()
        FillObjects()
        CalcArea()
        CalcIntensity(Image=Callose_Value, Total= yes)
        CalcAttr(AVG_Intensity, (intensity * 255)/area)
        Set(Intensity_Threshold = objects.AVG_Intensity.mean)
        ObjectFilter(AVG_Intensity > Intensity_Threshold * 0.525 && area > 5)
        object_contrast_general(reference = Callose_Value, ContrastDef = "WithoutCyto")
        ObjectFilter(contrast > 0.25 || AVG_Intensity > Intensity_Threshold * 1.25)
        set(ROI_Initial_Con=Objects)
        // Initial ROI objects have been detected

///*** Step 1.2: Detect ROI using Watershed ***///
        // This step can provide regions with bright pixels
        // using adaptive thresholding
        If(Callose_Value.mean / Callose_Value.median > 1.75) // image contains big intenstiy difference
                ThresholdXX(TuneTH=1.25, Image=Callose_Value)
                Mask(Threshold=Threshold * 1.95, Image=Callose_Value)
        Else() // a dark or clear image
                ThresholdXX(TuneTH=1.25, Image=Callose_Value)
                Mask(Threshold=Threshold * 2.15, Image=Callose_Value)
        End()
        Mask2Stencil()
        Stencil2Objects()

        // Use watershed method - this step is not necessary in case the input callose images are clear
        Maximums(2, Mask=objects.body.mask, Image=Callose_Value)
        Set(Points=Stencil2Objects(Stencil=Maximums).Objects)
        CalcWatershed(body, Objects=Points, Basins=objects.body, Image=Callose_Value, IntensityConstraintsMode=-1, MinBLC=0.05)
        Stencil2Objects(Objects.watershed)
        CalcArea()
        object_contrast_general(reference = Callose_Value, ContrastDef = "WithoutCyto")
        // get rid of small callose signals - there is no deposition
        CalcIntensity(Image=Callose_Value, Total= yes)
        CalcAttr(AVG_Intensity, (intensity * 255)/area)
```

```
		Set(Intensity_Threshold = objects.AVG_Intensity.mean)
		ObjectFilter(AVG_Intensity > Intensity_Threshold * 0.625 && contrast > 0.1 && area > 5)
		ObjectFilter(contrast > 0.25 || AVG_Intensity > Intensity_Threshold * 1.25)
		// Reassemble the area
		Mask2Stencil(objects.body.mask.image)
		Stencil2Objects()
		set(ROI_Initial_Watershed=Objects)

	///*** Step 1.3: Start to assemble the refined ROI ***///
		//*** Step 1.3.1: Assemble the ROI objects
		And(ROI_Initial_Watershed.body.mask.image, image = ROI_Initial_Con.body.mask.image)
		Or(ROI_Initial_BM.body.mask.image, image = image)
		// boolean operation "and" with initial the ROI mask
		Mask2Stencil(image)
		Stencil2Objects()
		FillObjects()
		CalcArea()
		object_contrast_general(reference = Callose_Value, ContrastDef = "WithoutCyto")
		ObjectFilter(contrast > 0.1 && area >= 5) // get rid of very low intensity and small objects
		if(RemoveBorderObj)
			RemoveBorderObjects(5)
		End()
		set(ROI_Refined_1 =Objects)
		// Finish finding the ROI objects - bright pixels are retained if they can be detected by both
		// the edge detection and the watershed

		//*** Step 1.3.2: Remove very big vessel/noise/hair signals
		ThresholdXX(TuneTH=1.25, Image=Callose_Value)
		Mask(Threshold=Threshold * 4.5, Image=Callose_Value)
		// Using thresholding to refine the edge detection of ROI objects
		Mask2Stencil()
		Stencil2Objects()
		FillObjects()
		CalcArea()
		CalcArea(border, AutoRecalc=yes)
		ObjectFilter(area > 325)
		CalcWidthLength(upto100 = no)
		object_contrast_general(reference = Callose_Value, ContrastDef = "WithoutCyto")
		ObjectFilter(full_length > 325 || ((full_length > 25 && full_length < 125) && (half_width > 5.25 && half_width < 10) && contrast > 0.625))

		// Filter based on shape and intensity
		CalcIntensity(Image=Callose_Value, Total=no, objects=objects)
		CalcAttr(Roundness, 4 * pi * (area / (border_area ^ 2)) * 100) // the Circularity field
		// Small hair or objects
		ObjectFilter((area < 925 && roundness > 30 && roundness < 65 && (intensity > Callose_Value.mean * 6.5 && contrast > 0.725)) || (area >= 925 && roundness < 30 && roundness > 9.5 && (intensity > Callose_Value.mean * 7.25 || contrast > 0.725)))

		Set(stencil = objects.body)
		CalcErosion(-3, objects = objects, Stencil = stencil) // increasing 3 pixels
```

```
            RenameAttr(body = stencil_eroded)
            Mask2Stencil(objects.body.mask.image)
            Stencil2Objects()
            Set(ROI_Refined_2 = objects)

            // Assemble the ROI objects list
            Xor(ROI_Refined_2.body.mask.image, image = ROI_Refined_1.body.mask.image)
            Mask2Stencil(image)
            Stencil2Objects()
            CalcArea()
            CalcWidthLength(Upto100=yes)
            Set(ROI_Refined_3 = objects)

        //*** Step 1.4: Start to remove high leaf vessel, mesophyll cells, and unsuitable objects
            If(ROI_Refined_3.area.max > 625 ||  ROI_Refined_3.full_length.max > 35) // find bright vessel or big noisy signals
                If(ROI_Refined_3.area.max > 2750 || ROI_Refined_3.full_length.max > 35)
                    // find very big/bright/long vessel or big noisy signals
                    // Divide the ROI objects according to their size/shape
                    ObjectFilter(area > 125 || full_length > 32.5, objects = ROI_Refined_3)
                    Set(ROI_Big_INI = objects)
                    ObjectFilter(area <= 125 && full_length <= 32.5, objects = ROI_Refined_3)
                    Set(ROI_Small_INI = objects)

                ///*** Step 1.4.1: Find refined small ROI ***///
                    CalcIntensity(Image=Callose_Value, Total=no, objects=ROI_Small_INI)
                    ObjectFilter(area > 5 && intensity > Callose_Value.mean * 1.5)
                    Set(ROI_Small_REF = objects)

                ///*** Step 1.4.2: Find refined big ROI ***///
                    ThresholdXX(TuneTH=1.25, Image=Callose_Value)
                    Mask(Threshold=Threshold * 2.15, Image=Callose_Value)
                    Mask2Stencil()
                    Stencil2Objects()
                    And(ROI_Big_INI.body.mask.image, image = objects.body.mask.image)
                    Mask2Stencil(image)
                    Stencil2Objects()
                    CalcArea()
                    CalcWidthLength(Upto100=yes)

                    If(!LargeCallose) // Large Callose deposits are not required
                        ObjectFilter(full_length > 0 && full_length < 25) // get rid of very big callose deposits
                    Else()
                        // Large callose deposits are rerquired
                        set(ROI_Big_INI_1 = objects)
                        // Divide the ROI objects list to handle very big objects
                        ObjectFilter(!(full_length <= 21.5 && area <125), objects = ROI_Big_INI_1)
                        set(ROI_Big_INI_1_Big = objects)
                        ObjectFilter(full_length <= 21.5 && area <125, objects = ROI_Big_INI_1)
                        set(ROI_Big_INI_1_Small = objects)
```

```
                ThresholdXX(TuneTH=1.25, Image=Callose_Value)
                Mask(Threshold=Threshold * 2.5, Image=Callose_Value)
                // Using thresholding to refine the edge detection of ROI objects
                Mask2Stencil()
                Stencil2Objects()
                And(ROI_Big_INI_1_Big.body.mask.image, image = objects.body.mask.image)
                Set(ROI_Big_INI_IMG = image)
                // Add relatvie small objects

                Or(ROI_Big_INI_1_small.body.mask.image, image = ROI_Big_INI_IMG)
                Mask2Stencil(image)
                Stencil2Objects()
                CalcArea()
                CalcWidthLength(Upto100=yes)
                ObjectFilter(area > 2.5 && !(full_length >375 && half_width > 12.5))
                CalcIntensity(Image=Callose_Value, Total=no, objects=objects)
                ObjectFilter(area > 5 && intensity > Callose_Value.mean * 1.5)
        End()
        Set(ROI_Big_REF = objects)

        // Not checking objects overlapping
        // Assemble small ROI and refined big ROI objects
        Or(ROI_Small_REF.body.mask.image, image=ROI_Big_REF.body.mask.image)
        Mask2Stencil(image)
        Stencil2Objects()
        CalcArea()
        CalcWidthLength(Upto100=yes)
        Set(ROI_Refined_INI = objects)

Else() // Cannot find very big ROI objects
        // Divide objects list into two groups
        ObjectFilter(area > 125, objects = ROI_Refined_3)
        Set(ROI_Big_INI = objects)
        ObjectFilter(area <= 125, objects = ROI_Refined_3)
        Set(ROI_Small_INI = objects)

        ///*** Step 1.4.1: Find refined small ROI ***///
        CalcIntensity(Image=Callose_Value, Total=no, objects=ROI_Small_INI)
        ObjectFilter(area > 5 && intensity > Callose_Value.mean * 1.5)
        Set(ROI_Small_REF = objects)

        ///*** Step 1.4.2: Find refined Big ROI Objects ***///
        ThresholdXX(TuneTH=1.25, Image=Callose_Value)
        Mask(Threshold=Threshold * 1.75, Image=Callose_Value)
        Mask2Stencil()
        Stencil2Objects()
        And(ROI_Big_INI.body.mask.image, image = objects.body.mask.image)
        Mask2Stencil(image)
```

```
                Stencil2Objects()
                CalcArea()
                CalcWidthLength(Upto100=yes)
                ObjectFilter(area > 2.5 && !(full_length >325 && half_width > 10))
                CalcIntensity(Image=Callose_Value, Total=no, objects=objects)
                ObjectFilter(area > 5 && intensity > Callose_Value.mean * 1.5)
                Set(ROI_Big_REF = objects)

                // Not checking objects overlapping
                // Assemble small ROI and refined big ROI objects
                Or(ROI_Small_REF.body.mask.image, image=ROI_Big_REF.body.mask.image)
                Mask2Stencil(image)
                Stencil2Objects()
                CalcArea()
                CalcWidthLength(Upto100=yes)
                Set(ROI_Refined_INI = objects) // Use "ROI_refined" as the output objects list for this stage
        End()
Else()
        Set(ROI_Refined_INI = ROI_Refined_3)
End()

// Get rid of small objects if required - certain experiments only need to detect big callose deposition
If(!SmallCallose)
        ObjectFilter(area > 10 && (full_length > 2.5 || half_width > 1.25), objects = ROI_Refined_INI)
        set(ROI_Refined_INI = objects)
End()

///*** Step 1.4.3: categorise ROI Objects ***///
ObjectFilter(!(full_length > 10 && half_width < 1.5), objects = ROI_Refined_INI)
object_contrast_general(reference = Callose_Value, ContrastDef = "WithoutCyto")
CalcArea()
CalcArea(border)
CalcAttr(Roundness, 4 * pi * (area / (border_area ^ 2)) * 100) // the Circularity field
CalcIntensity(Image=Callose_Value, Total= no)
CalcWidthLength(Upto100= no)
set(TMP_ROI_Refined_INI = objects)

// Leave vessel or long fused callose
ObjectFilter((intensity < 0.8 * Callose_Value.max && intensity > 0.2 * Callose_Value.max) && ((full_length > 20 && half_width < 7.25 && contrast < 0.65) || (full_length > 50 && half_width < 10.25 && contrast < 0.75) || (full_length > 92.5 && half_width < 13.25 && contrast < 0.85)), objects = TMP_ROI_Refined_INI)
Set(ROI_Refined_Vessel = Objects)

// Remove Vessel
CalcStat("mean", Stencil = TMP_ROI_Refined_INI.body, Image = ROI_Refined_Vessel.body.mask.image, AttrName="overlapped", objects=TMP_ROI_Refined_INI)
ObjectFilter(overlapped == 0, objects = objects )
ObjectFilter(roundness > 30 || (contrast > 0.35 && intensity > Callose_Value.max * 0.5) || area > 1250)
ObjectFilter(!(intensity > Callose_Value.max * 0.75 && contrast > 0.825))
ObjectFilter(!(intensity > Callose_Value.max * 0.725 && contrast > 0.775 && half_width > 8.5 && (roundness < 70 && roundness > 35) && area > 375 && full_length > 25))
// Remove Hairs
```

```
		Set(ROI_Refined = objects)
		// Include fused Callose and seperated callose
///*** Finish Detecting ROI and generating ROI objects list***///


///*** STEP TWO: Start to Detect Callose Deposits Based on ROI ***///
  //** Step 2.1: Detect centres of callose candidates based on ROI
		// Detect Zone image and centres of Callose Deposits
		// Finds the maximum intensity pixels within ROI.
		if(ROI_BIG_REF.area.mean > 150) // Contain very large ROI objects
			Maximums(Distance= 2, Mask=ROI_Refined.Body, Image=Callose_IMG) // Scan 2-pixel regions
		Else()
			Maximums(Distance= 3, Mask=ROI_Refined.Body, Image=Callose_IMG) // Scan 3-pixel regions
		End()
		// do not use zone image as it generates imprecise centres

		Set(stencil = maximums)
		Stencil2Objects()
		CalcIntensity(Image=Callose_Value, Total= no)
		Set(Intensity_Threshold = Callose_Value.mean)
		ObjectFilter(Intensity > Intensity_Threshold * 2.25) // Only retain points with very high intensity values
		Set(Callose_Cadidates_Centre = objects)

		// Start to combine some of the miss separated callose centres
		Set(stencil = objects.body)
		// pi * 3.5 * 3.5 = 38.5 - the size of small callose deposits is bigger than 40 based on median value
			CalcErosion(-1, objects = objects, Stencil = stencil) // increasing 1 pixel
		// Small callose objects are relatively small

		RenameAttr(body = stencil_eroded)
		Mask2Stencil(objects.body.mask.image)
		Stencil2Objects()
		CalcIntensity(Image=Callose_Value, Total= yes)
		CalcArea()
		CalcAttr(AVG_Intensity, intensity/area) // Apply local thresholding
		Set(Intensity_Threshold = Callose_Value.mean)
		ObjectFilter(AVG_Intensity > Intensity_Threshold * 2.5) // get as many centres as possible
		CalcWidthLength(Upto100=yes) // get the centres of the body
		Set(Callose_Cadidates_Centre_refined = objects) // Refined detection on callose centres!!!!!

		//** Step 2.1.1: Detect centres of very big callose candidates based on ROI
		If(ROI_BIG_REF.area.mean > 150)
		// image contains very big callose
		// Apply global thresholding
			ThresholdXX(TuneTH=1.25, Image=Callose_Value)
			Mask(Threshold=Threshold * 5.525, Image=Callose_Value)
		Else()
			ThresholdXX(TuneTH=1.25, Image=Callose_Value)
			Mask(Threshold=Threshold * 5.325, Image=Callose_Value)
```

```
        End()
        Mask2Stencil()
        Stencil2Objects()
        CalcArea()
        CalcWidthLength(Upto100=yes)
        ObjectFilter(area > 75 && half_width > 3.75 && full_length > 9.25)
        ObjectFilter(!(area > 1250 && half_width > 8.25 && full_length > 95))
        set(Big_Callose_Area = objects)

        If(Big_Callose_Area.@count > 0)
            CalcStat("mean", Stencil = Callose_Cadidates_Centre_refined.body, Image = Big_Callose_Area.body.mask.image, AttrName="overlapped", objects=Callose_Cadidates_Centre_refined)
            ObjectFilter(overlapped == 0, objects = objects )
            Set(Callose_Cadidates_Centre_refined_Small = objects) // Finish filtering the centres for big callose

            Maximums(Distance= 4.5, Mask=Big_Callose_Area.Body, Image=Callose_IMG) // Scan 4.5-pixel regions
            Set(stencil = maximums)
            Stencil2Objects()
            CalcIntensity(Image=Callose_Value, Total= no)
            Set(Intensity_Threshold = Callose_Value.mean)
            ObjectFilter(Intensity > Intensity_Threshold * 2.15) // Only retain points with very high intensity values
            Set(Callose_Cadidates_Centre_refined_Big = objects)

            Set(stencil = Callose_Cadidates_Centre_refined_Small.center_spot)
            Stencil2Objects()
            Set(Callose_Cadidates_Centre_refined_Small_centres = objects)
            AddObjects(Callose_Cadidates_Centre_refined_BIG, objects=Callose_Cadidates_Centre_refined_Small_centres, CheckOverlap=no, DeleteGeometry=no)

            Set(stencil = objects.body)
            CalcErosion(-1, objects = objects, Stencil = stencil) // increasing 1 pixel
            // Small callose objects are relatively small
            RenameAttr(body = stencil_eroded)
            Mask2Stencil(objects.body.mask.image)
            Stencil2Objects()
            CalcWidthLength(Upto100=yes) // get the centres of the body
            Set(Callose_Cadidates_Centre_refined = objects) // Refined detection on callose centres!!!!!
        End()
        // Finish refining the callose centres


    //** Step 2.2: Finalise callose candidates based on refined the centres
        Or(Callose_Cadidates_Centre_refined.body.mask.image, image = ROI_Refined.body.mask.image)
        Mask2Stencil(image)
        // Replace very small callose with eroded callose centres!!! important for rectifying the detected objects
        ObjectsFromCenters(Centers=Callose_Cadidates_Centre_refined.center_spot, RestrictiveStencil=stencil, image=Callose_Value, MinimumArea= 5)
        ControlBreakingLines_A(1) // Split as many objects as possible
        FillObjects()
        CalcArea(objects=objects, AutoRecalc=yes)
        ObjectFilter(area > 5)
```

```
        object_contrast_general(reference = Callose_Value, ContrastDef = "WithoutCyto")
        CalcIntensity(Image=Callose_Value, Total= yes)
        CalcAttr(AVG_Intensity, (intensity * 255)/area) // Apply local thresholding
        Set(Intensity_Threshold = Callose_Value.mean)
        ObjectFilter(AVG_Intensity > Intensity_Threshold * 3.5 && contrast > 0.025) // get as many centres as possible
        CalcWidthLength(Upto100=yes)
        CalcAttr(WLRatio, half_width * 2 / full_length)
        ObjectFilter(!(area > 25 && WLRatio < 0.165))
        ObjectFilter(!(area > 225 && WLRatio < 0.5 && AVG_Intensity/255 > Callose_Value.max * 0.925 ))
        Set(Callose_Candidates_final = objects)
        // Finish finding the candidates of callose deposits


//** Step 2.3: Perform Edge Detection on Callose Candidates
        // Divide Callose candidates according to their sizes
        Set(Callose_Initial = Callose_Candidates_final)
        If(!LargeCallose) // pi * 3.5 * 3.5 = 38.5
                ObjectFilter(area > 125 && area <= 500, objects = Callose_Candidates_final)
        Else()
                ObjectFilter(area > 125 && area <= 1250, objects = Callose_Candidates_final)
                // some experiments can generate very big callose
        End()
        Set(Callose_Initial_Big = objects)

        If(!SmallCallose)
                ObjectFilter(area <= 125 && area >= 10, objects = Callose_Candidates_final)
        Else()
                ObjectFilter(area <= 125 && area >= 5, objects = Callose_Candidates_final)
        End()
        Set(Callose_Initial_Small = objects)
        //// Finish categorising callose candidates according to their size

        //** Step 2.3.1: Perform Edge-based Detection on Big Callose Deposition and Remove Experimental Errors
        EdgeDetection_BIG(Callose_Value, Callose_Cadidates_Centre_refined, Callose_Initial_Big, ROI_Refined)
        //*** Finish refining big callose deposits

        //*** Step 2.3.2: Start to process small callose
        // Start to refine the outline of small callose (big)
        EdgeDetection_SMALL(Callose_Value, Callose_Cadidates_Centre_refined, Callose_Initial_Small, ROI_Refined)
        //*** Finish refining small callose deposits


        //*** Step 2.3.3: Start to process small callose on plant vessel
        // Start to refine the outline of small callose (big)
        // In the future, another class can be added to detect callose with size below 50 pixel2
        If(ROI_Refined_Vessel.intensity.mean > 0.25)
        // image contains big intenstiy difference
        // Apply global thresholding
                ThresholdXX(TuneTH=1.25, Image=Callose_Value)
```

```
		Mask(Threshold=Threshold * 3.85, Image=Callose_Value)
	Else()
		ThresholdXX(TuneTH=1.25, Image=Callose_Value)
		Mask(Threshold=Threshold * 3.75, Image=Callose_Value)
	End()
	Mask2Stencil()
	Stencil2Objects()
	set(Brightest_Area_Ini_Vessel = objects)

	// Refine the ROI on plant vessel
	And(Brightest_Area_Ini_Vessel.body.mask.image, image = ROI_Refined_Vessel.body.mask.image)
	Mask2Stencil(image)
	Stencil2Objects()
	CalcArea()
	CalcWidthLength(Upto100= no)
	CalcAttr(WL_Ratio, full_length/(half_width * 2))
	CalcIntensity(Image=Callose_Value, Total= no)
	object_contrast_general(reference = Callose_Value, ContrastDef = "WithoutCyto")
	ObjectFilter((WL_Ratio <= 3.75 && half_width > 4.5 && full_length > 27.5) || area < 325 || WL_Ratio > 7.5 || (WL_Ratio > 4.5 && area > 975) || (WL_Ratio > 3.75 && half_width < 7.5 && full_length > 27.5 ))
	ObjectFilter(!(area > 425 && intensity > Callose_Value.max * 0.775 && contrast > 0.725))
	set(Callose_Vessel_INI = objects)

	if(Callose_Vessel_INI.area.mean > 32.5) // Contain large ROI objects
		Maximums(Distance= 4, Mask=Callose_Vessel_INI.Body, Image=Callose_IMG) // Scan 3-pixel regions
	Else()
		Maximums(Distance= 3, Mask=Callose_Vessel_INI.Body, Image=Callose_IMG) // Scan 2-pixel regions
	End()
	// do not use zone image as it generates imprecise centres

	Set(stencil = maximums)
	Stencil2Objects()
	CalcIntensity(Image=Callose_Value, Total= no)
	Set(Intensity_Threshold = ROI_Refined_Vessel.intensity.mean)
	ObjectFilter(Intensity > Intensity_Threshold * 1.25) // Only retain points with very high intensity values
	Set(Callose_Vessel_Centres = objects)

	// Start to detect callose on vessel
	ObjectsFromCenters(Centers=Callose_Vessel_Centres.body, RestrictiveStencil=Callose_Vessel_INI.body, image=Callose_Value, MinimumArea= 5)
	ControlBreakingLines_A(1)
	Set(stencil = objects.body)
	Stencil2Objects()
	CalcArea()
	CalcIntensity(Image=Callose_Value, Total= no)
	ObjectFilter(Intensity > Intensity_Threshold && area > 2.5)
	object_contrast_general(reference = Callose_Value, ContrastDef = "WithoutCyto")
	ObjectFilter(contrast > 0.325 || Intensity > Intensity_Threshold * 1.525 )
	ObjectFilter(!(intensity > Callose_Value.max * 0.9 && contrast > 0.65 && area > 125))
	ObjectFilter(!(intensity > Callose_Value.max * 0.825 && contrast > 0.625 && area > 275))
```

```
CalcWidthLength(Upto100=yes)
ObjectFilter(half_width >= 1 && full_length >= 2)
CalcAttr(WL_Ratio, full_length/(half_width * 2))
ObjectFilter(WL_Ratio <= 3.5)
ObjectFilter(WL_Ratio <= 2.75 || (Intensity_Threshold * 1.65 && contrast > 0.35)) // exceptional cases
// get as many callose deposits as possible
Set(Callose_Vessel_Refined = objects)


// Refine the centre detection with vessel centers
Set(stencil = Callose_Vessel_Refined.center_spot)
Stencil2Objects()
CalcErosion(-2, objects = objects, Stencil = stencil) // increasing 2 pixels
RenameAttr(body = stencil_eroded)

// Add vessl centres to the callose center objects list
Or(objects.body.mask.image, image = Callose_Cadidates_Centre_refined.body.mask.image)
Mask2Stencil(image)
Stencil2Objects()
CalcWidthLength(Upto100=yes)
Set(Callose_Cadidates_Centre_final = objects)
//*** Finish refining callose centers with centres on vessel

//** Step 2.4: Start to assemble the detected big/small callose objects list
Or(Callose_Small_refined.body.mask.image, image = Callose_Big_refined.body.mask.image)
Or(image, image = Callose_Vessel_Refined.body.mask.image)
Mask2Stencil(image)
Stencil2Objects()
Set(Callose_Candidates_refined =objects)

// Refine object centres for detected callose objects
ObjectsFromCenters(Callose_Cadidates_Centre_final.center_spot, Callose_Candidates_refined.body, Image=Callose_Value, MinimumArea= 4)  // still use 4 pixel square as the
Minimum Area
ControlBreakingLines_A(1) // Split objects as many as possible

Set(stencil = objects.body)
Stencil2Objects()
CalcBorder(AutoRecalc=yes, objects=objects)
CalcArea(AutoRecalc=yes, objects=objects)
CalcArea(border, AutoRecalc=yes, objects=objects)

// Get rid of small callose if required
If(!SmallCallose)
        ObjectFilter(area >= 25 && border_area >= 8)
Else()
        ObjectFilter(area >= 5 && border_area >= 4)
End()

if(RemoveBorderObj)
```

```
		RemoveBorderObjects(25)
End()

// Start to screen out fake callose objects
CalcWidthLength(Upto100=yes)
ObjectFilter(half_width > 1 && full_length >= 2 && full_length < 42.5)
CalcIntensity(Image=Callose_Value, Total=No, CalcStdDev=no)
CalcIntensity(border, Callose_Value, Total=No, CalcStdDev=no)

CalcAttr(Intensity_Diff, (intensity - border_intensity)/(intensity + border_intensity))
set(Intensity_Threshold = objects.intensity.mean)
ObjectFilter(intensity_Diff > 0 || intensity > Callose_Value.mean * 3.25)
ObjectFilter((area >= 32.5 && (Intensity_Diff > 0.03 || intensity > Intensity_Threshold * 1.125)) || (area < 32.5 && (Intensity_Diff > 0.02 || intensity > Intensity_Threshold)))
// Use global intensity threshold
object_contrast_general(reference = Callose_Value, ContrastDef = "WithoutCyto")
ObjectFilter(contrast > 0.275 || (area < 50 && contrast > 0.25) || (intensity > Callose_Value.mean * 3.5 && Intensity_Diff > 0.025 && contrast > 0.185))
// Filter based on contrast, intensity, and the intensity of callose centres

If(Callose_Value.mean > 0.125)
		ObjectFilter(Intensity_Diff > 0.025 || intensity > Callose_Value.mean * 3.05 || contrast > 0.25)
Else()
		ObjectFilter(Intensity_Diff > 0.025 || intensity > Callose_Value.mean * 2.75 || contrast > 0.225)
End()
Set(Intensity_Threshold = objects.Intensity.mean) // Use loacl intensity threshold

// Filter based on contrast and intensity
If(LargeCallose)
		ObjectFilter(Intensity > Intensity_Threshold * 0.725 || contrast > 0.175)
Else()
		ObjectFilter(Intensity > Intensity_Threshold * 0.625 || contrast > 0.155)
		ObjectFilter(!(area > 50 && (contrast < 0.525 || Intensity > Intensity_Threshold * 1.35)))
End()

If(!SmallCallose)
		ObjectFilter(area > 20 || contrast > 0.275 || Intensity > Intensity_Threshold * 0.95)
Else()
		ObjectFilter(area > 10 || contrast > 0.225 || Intensity > Intensity_Threshold * 0.75)
End()

CalcAttr(Roundness, 4 * pi * (area / (border_area ^ 2)) * 100) // the Circularity field
CalcAttr(WL_Ratio, full_length/(half_width * 2))
ObjectFilter(WL_Ratio < 4) // get rid of flat objects

// Filter based on roundness and width and length
If(LargeCallose)
		ObjectFilter((WL_Ratio < 3.85 && Roundness > 47.5) || area < 40)
		// some strange shape can be detected based on objects split due to centres cannot be separated
		ObjectFilter((WL_Ratio < 2.5 && Roundness > 110) || contrast >= 0.225 || area < 40 || intensity > Callose_Value.mean * 3.75)
Else()
```

```
                ObjectFilter(WL_Ratio < 3.75 && Roundness > 50 || area < 47.5)
                ObjectFilter((WL_Ratio < 2.5 && Roundness > 110) || contrast >= 0.25 || area < 40 || intensity > Callose_Value.mean * 4.25)
            End()

            // Exceptional case
            If(!LargeCallose)
                ObjectFilter(!(full_length >13.75 || half_width > 5 || (WL_Ratio > 2 && area > 125)))
            End()
            ObjectFilter(!(WL_Ratio > 2.25 && intensity < Callose_Value.mean))
            ObjectFilter(!(WL_Ratio > 2.25 && area > 50 && intensity < Callose_Value.mean * 4.25) || !(WL_Ratio > 2.25 && area > 50 && contrast < 0.275))
            ObjectFilter(!(area > 75 && (Intensity_Diff < 0.05 && intensity < Callose_Value.mean * 4.25)))
            Set(Callose_refined_final = objects)



        ///*** Optional Functions ***///
        ///** START TO DETECT SPREADING CALLOSE NETWORKS **///
            If(Callose_Network_Trigger && Callose_refined_final.@count > 1)
        ///*** Step Three: Start to calculate the callose network  ***///
                Set(DrawLine_Callose_IMG = Callose_Value)
                set(IM_Height_Expanded = Callose_Value.height)
                set(IM_Width_Expanded =  Callose_Value.width)
                Blank(IM_Width_Expanded, IM_Height_Expanded, 0)
                set(Callose_Network_IMG = image) // Create a blank image

                If(Network_Threshold == 25) // the default value, the distance threshold is calculated below
                    If(Callose_refined_final.full_length.mean > Callose_refined_final.full_length.median)
                        set(Network_Threshold = (Callose_refined_final.full_length.mean + Callose_Big_refined.full_length.mean +
Callose_Small_refined.full_length.mean))
                        If(Network_Threshold < 20 || Network_Threshold > 27.5)
                            set(Network_Threshold = 22.5)
                        End()
                    Else()
                        set(Network_Threshold = (Callose_refined_final.full_length.median + Callose_Big_refined.full_length.median +
Callose_Small_refined.full_length.median))
                        If(Network_Threshold < 20 || Network_Threshold > 27.5)
                            set(Network_Threshold = 22.5)
                        End()
                    End()
                End()

            //** Step 3.1: Detect the callose network
                Foreach(0..(Callose_refined_final.@count - 1), Obj_Counter)
                    Set(Current_Centre_Spot = Obj_Counter)
                    set(Obj_Distance_Counter = 0)
                    Set(current_X_Axis = Callose_refined_final.center_spot.x.target[Current_Centre_Spot])
                    Set(current_Y_Axis = Callose_refined_final.center_spot.y.target[Current_Centre_Spot])

                    ///Determine the area of the detection
```

```
				Mask2Stencil(Callose_refined_final[Current_Centre_Spot].body.image)
				Stencil2Objects()
				CalcErosion(Network_Threshold * -1, objects = objects, Stencil = stencil) // increasing 1 pixel
				RenameAttr(body = stencil_eroded)
				Set(Temp_Callose_Scan_Obj = Callose_refined_final)

				CalcStat("mean", Stencil = Temp_Callose_Scan_Obj.body, Image = objects.body.mask.image, AttrName="overlapped", objects=Temp_Callose_Scan_Obj)
				ObjectFilter(overlapped != 0, objects = objects )
				Set(Temp_Callose_Scan_Obj = objects)

				Foreach(0..(Temp_Callose_Scan_Obj.@count - 1), Obj_Distance_Counter)
					If(Temp_Callose_Scan_Obj.center_spot.x.target[Obj_Distance_Counter] != current_X_Axis && Temp_Callose_Scan_Obj.center_spot.y.target[Obj_Distance_Counter] != current_Y_Axis)
						// Calculate the distance
						// Set(Callose_Distance = sqrt((current_X_Axis - Temp_Callose_Scan_Obj.center_spot.x.target[Obj_Distance_Counter])^2 + (Temp_Callose_Scan_Obj.center_spot.y.target[Obj_Distance_Counter] - current_Y_Axis)^2))

						DrawLine(point(current_X_Axis, current_Y_Axis), point(Temp_Callose_Scan_Obj.center_spot.x.target[Obj_Distance_Counter], Temp_Callose_Scan_Obj.center_spot.y.target[Obj_Distance_Counter]), color="white", image=DrawLine_Callose_IMG)
						Set(DrawLine_Callose_IMG = image)

						DrawLine(point(current_X_Axis, current_Y_Axis), point(Temp_Callose_Scan_Obj.center_spot.x.target[Obj_Distance_Counter], Temp_Callose_Scan_Obj.center_spot.y.target[Obj_Distance_Counter]), "intensity", 215, "yellow", image=Callose_Network_IMG)
						// generate LINKS with DETERMINED coordinates
						OR(image, image = Callose_Network_IMG)
						set(Callose_Network_IMG = image)
					End()
				End()
			End()

		//** Step 3.2: Calculate the area of the callose network
			Mask2Stencil(Callose_Network_IMG)
			Stencil2Objects()
			CalcErosion(-1, objects = objects, Stencil = stencil) // increasing 1 pixel
			RenameAttr(body = stencil_eroded)
			Mask2Stencil(objects.body.mask.image)
			Stencil2Objects()
			Set(Callose_Network = objects)

			Mask2Stencil(Callose_Network.body.mask.image)
			Stencil2Objects()
			FillSmallHoles(150)
			CalcWidthLength()
			CalcArea()
			CalcArea(border, objects = objects)
			ObjectFilter(full_length > Network_Threshold * 1.75 && (half_width > 2.75 || full_length > Network_Threshold * 2.25 ) && area > 125)
			Set(Callose_Network_refined = objects)

		//** Step 3.3: Detect the skeleton of the network based on intensity of objects
```

```
                    CalcSkeletonByIntensity(SkeletonType="cornerconnected", Image=Callose_Value, IntensityEvalParam=-oo, Objects=Callose_Network_refined)
                    set(stencil = objects.skeleton)
                    Stencil2Objects()
                    CalcArea(AutoRecalc=yes)
                    CalcIntensity(Image=Callose_Value, CalcStdDev=yes, Total=yes)

                    Mask2Stencil(objects.body.mask.image)
                    Stencil2Objects()
                    CalcErosion(-2, objects = objects, Stencil = stencil) // increasing 2 pixels
                    RenameAttr(body = stencil_eroded)
                    Mask2Stencil(objects.body.mask.image)
                    Stencil2Objects()
                    CalcArea()
                    CalcArea(border, objects = objects)
                    CalcWidthLength()
                    Set(Callose_Network_Skeleton = objects)
            End()
            ///** Callose Network Construction Finishes **///


    ///*** Step Four: Start to assemble output fields ***///
            // output generated images
            set( namelength = length(Source_Path) )
            set( corename = substr( Source_Path, 1, namelength - 4))

     //** Step 4.1:Preview images if required
            If(ShowIllustrations)
                    imageview(Callose_refined_final.body, "Detected_Callose", image=Callose_IMG, middlecolor=rgb(0x00,0xff,0x00))
                    If(Callose_Network_Trigger)
                            imageview(Callose_Network.body, "Callose_Network", image=Callose_IMG, middlecolor=rgb(0x00,0xff,0x00))
                            imageview(Callose_Network_Skeleton.body, "Callose_Network", image=Callose_IMG, middlecolor=rgb(0x00,0xff,0x00))
                    End()
            End()

     //** Step 4.2: Export result images
            Gamma(1.0, image=Callose_IMG)
            WriteImage(imagefile=CreateDir & "/" & Experiment_Name & "_1_Original_IMG.png", image=Image, imageformat="png")

            Gamma(1.0, image=Callose_IMG)
            CarryObjects(Callose_refined_final.body, image.max)
            CarryObjects(Callose_refined_final.body, "rainbow")
            WriteImage(imagefile=CreateDir & "/" & Experiment_Name & "_2_Detected_Callose_Obj.png", image=Image, imageformat="png")

            set(stencil = Callose_refined_final.body)
            Stencil2Objects()
            // enlarge border stencil by 1 pixel for better visualisation
            Gamma(1.0, image=Callose_IMG)
            CarryObjects(objects.border, image.max)
            CarryObjects(objects.border, "green")
```

```
		WriteImage(imagefile=CreateDir & "/" & Experiment_Name & "_3_Detected_Callose_Outline.png", image=Image, imageformat="png")

		ObjectNumberOnImage(Callose_refined_final, "yellow", 14, image=Callose_IMG)
		WriteImage(imagefile=CreateDir & "/" & Experiment_Name & "_4_Labelled_Callose.png", image=Image, imageformat="png")

		If(Callose_Network_Trigger)
			Gamma(1.0, image=Callose_IMG)
			CarryObjects(Callose_Network.body, image.max)
			CarryObjects(Callose_Network.body, "red")
			WriteImage(imagefile=CreateDir & "/" & Experiment_Name & "_5_Callose_Network.png", image=Image, imageformat="png")

			Gamma(1.0, image=Callose_IMG)
			CarryObjects(Callose_Network_Skeleton.body, image.max)
			CarryObjects(Callose_Network_Skeleton.body, "red")
			WriteImage(imagefile=CreateDir & "/" & Experiment_Name & "_6_Spreading_Skeleton.png", image=Image, imageformat="png")
		End()

	//** Step 4.3: Export overall result CSV file
		If(Callose_refined_final.@count > 1)
			printfopen(CreateDir & "/" & Output_File & ".csv", yes)
			Printf(Experiment_Name & "#")
			Printf(Callose_refined_final.@count & "#")
			Printf(Callose_refined_final.area.mean & "#")
			Printf(Callose_refined_final.Intensity.mean*255 & "#")
			// Network is required
			If(Callose_Network_Trigger) // change as not ticking callose network will cause error
				If(Callose_Network_refined.@count >0)
					Delete(Network_Length)
					Set(Network_Length = ((Callose_Network_Skeleton.border_area.sum - Callose_Network_Skeleton.half_width.sum * 2)/2))
					Printf(Network_Length & "#") // the calculated network length
				Else()
					Printf(nan & "#")
				End()
			End()
			Printf(Callose_refined_final.area.sum & "#\n") // the measured area of callose deposits in pixels square
			printfopen()
		Else()
			printfopen(CreateDir & "/" & Output_File & ".csv", yes)
			Printf(Experiment_Name & "#")
			Printf( 0 & "#")
			Printf(nan & "#")
			Printf(nan & "#")
			// Network is required
			If(Callose_Network_Trigger)
				Printf(nan & "#")
			End()
			Printf(nan & "#\n")
			printfopen()
		End()
```

```
                // Close the csv writing

        //** Step 4.4: Start to export individual callose
                set(Callose_Counter = 0)
                If(Callose_refined_final.@count > 1)
                        Foreach(0..(Callose_refined_final.@count-1), Callose_Counter)
                                // Start to build up the output table
                                printfopen(CreateDir & "/" & Experiment_Name & "_Callose_Results"& ".csv", yes)
                                Printf(Experiment_Name & "#")
                                Printf((Callose_Counter + 1) & "#")
                                Printf(Callose_refined_final.area[Callose_Counter] & "#")
                                Printf(Callose_refined_final.Roundness[Callose_Counter]/100 & "#")
                                Printf(Callose_refined_final.Intensity[Callose_Counter] * 255 & "#\n")
                                printfopen()
                        End()
                Else()
                        // Start to build up the output table
                        printfopen(CreateDir & "/" & Experiment_Name & "_Callose_Results"& ".csv", yes)
                        Printf(Experiment_Name & "#")
                        Printf( 0 & "#")
                        Printf(nan & "#")
                        Printf(nan & "#")
                        Printf(nan  & "#\n")
                        printfopen()
                End()

                // finish preparing the output csv file
                Printf("Finish analysing image %s \n", files[file_counter])
        end()
        ///** Finish analysing one image **///
    End()
End()
///** Finish analysing the whole folder **///
```

```
//*** Internal Functions ***//
proc FillSmallHoles(
int minimumholearea=20 in "Minimum area for holes. Holes with area less than the limit are filled",
objectlist objects inout "Input-output object list. Attributes in the input list are lost."
) "Fills small holes in objects. Holes with area less than the limit MinimumHoleArea are filled."
{
        set(obj_in=objects)
        fillobjects()
        set(obj01=objects)
        carrypixels(image=obj01.body.image, mask=obj_in.body.mask, data=0)
        stencil2mask(image.vector)
        mask2stencil()
        stencil2objects()
        calcarea()
        objectfilter(minimumholearea<=area)

        carrypixels(image=obj01.body.image,mask=objects.body.mask,data=0)
        stencil2objects(image.vector)
        xor(image=objects.body.image,mask=obj_in.body.mask.image)
        setattr(filled, image.vector)
}


proc EdgeDetection_BIG(
image Callose_Image in "the input callose image",
objectlist Callose_Centres in "Input objects list for Callose centers.",
objectlist Callose_objects in "Input objects list for Callose candidates.",
objectlist ROI_Refined in "Input objects list which defines the ROI.",
objectlist Callose_Big_refined out "Output objects list with refined edge detection."
) //Robatzek Procedures "Perform the edge detection using edge tracing and adaptive thresholding."
{
        If(Callose_Image.stddev > 0.125)
                // image contains big intenstiy difference
                // Apply global thresholding
                ThresholdXX(TuneTH=1.25, Image=Callose_Image)
                Mask(Threshold=Threshold * 4.15, Image=Callose_Image)
        Else()
                ThresholdXX(TuneTH=1.25, Image=Callose_Image)
                Mask(Threshold=Threshold * 3.95, Image=Callose_Image)
        End()

        Mask2Stencil()
        Stencil2Objects()
        set(Brightest_Area_Ini_Big = objects)

        And(Brightest_Area_Ini_Big.body.mask.image, image = ROI_Refined.body.mask.image)
        And(Callose_objects.body.mask.image, image = image)
        Set(Callose_ROI_BIG = image)
        Mask2Stencil(Callose_ROI_BIG)
```

```
set(restrict_stencil = stencil)

Stencil2Objects(restrict_stencil)
CalcArea(AutoRecalc=yes, objects=objects)
object_contrast_general(reference = Callose_Image, ContrastDef = "WithoutCyto")
CalcIntensity(Image=Callose_Image, Total= no)
Set(Intensity_Threshold = objects.intensity.mean) // Use local thresholding
ObjectFilter(contrast > 0.25 || intensity > Intensity_Threshold * 0.5)
Set(Callose_Initial_Big_processed = objects)

// Divide the big callose into two groups
ObjectFilter(area > 50, objects = Callose_Initial_Big_processed)
Set(Callose_Initial_Big_processed_Big = objects)
ObjectFilter(area <= 50 && area >= 5, objects = Callose_Initial_Big_processed)
Set(Callose_Initial_Big_processed_Small = objects)

// Start to process small objects in big callose deposits
// Get rid of furry edges
Set(stencil = Callose_Initial_Big_processed_Small.body)
CalcErosion(-1, objects = Callose_Initial_Big_processed_Small, Stencil = stencil) // increasing 1 pixel
RenameAttr(body = stencil_eroded)
Set(stencil = objects.body)
CalcErosion(1, objects = objects, Stencil = stencil) // decreasing 1 pixel
RenameAttr(body = stencil_eroded)
Mask2Stencil(objects.body.mask.image)
Stencil2Objects()
CalcArea()
CalcIntensity(Image=Callose_Image, Total= yes)
CalcAttr(AVG_Intensity, (intensity/area)) // Apply local thresholding
Set(Intensity_Threshold = Callose_Image.mean)
ObjectFilter(AVG_Intensity > Intensity_Threshold * 2.75 && area > 5) // get as many centres as possible
Set(Callose_Initial_Big_processed_Small = objects)

// Start to process big objects in the big callose deposits
// Get rid of furry edges
If(Callose_Initial_Big_processed_Big.intensity.mean < Callose_Image.max * 0.75)
	If(Callose_Image.stddev > 0.125) // image contains big intensity difference
		ThresholdXX(TuneTH=1.25, Image=Callose_Image)
		Mask(Threshold=Threshold * 4.525, Image=Callose_Image)
	Else()
		ThresholdXX(TuneTH=1.25, Image=Callose_Image)
		Mask(Threshold=Threshold * 4.325, Image=Callose_Image)
	End()

	Mask2Stencil()
	Stencil2Objects()
	// Refine the edge detection of relatively big callose
	And(Objects.body.mask.image, image = Callose_Initial_Big_processed_Big.body.mask.image)
Else()
```

```
                Set(image = Callose_Initial_Big_processed_Big.body.mask.image)
        End()

        Mask2Stencil(image)
        Stencil2Objects()
        CalcErosion(-1, objects = objects, Stencil = stencil) // increasing 1 pixel
        RenameAttr(body = stencil_eroded)
        Set(stencil = objects.body)
        CalcErosion(1, objects = objects, Stencil = stencil) // decreasing 1 pixel
        RenameAttr(body = stencil_eroded)

        Mask2Stencil(objects.body.mask.image)
        Stencil2Objects()
        CalcArea()
        CalcIntensity(Image=Callose_Image, Total= yes)
        CalcAttr(AVG_Intensity, (intensity/area)) // Apply local thresholding
        Set(Intensity_Threshold = Callose_Image.mean)
        ObjectFilter(AVG_Intensity > Intensity_Threshold * 2.75) // get as many centres as possible
        Set(Callose_Initial_Big_processed_Big = objects)

        // Rebuild the callose objects list based on the refined callose centres
        Or(Callose_Initial_Big_processed_Small.body.mask.image, image = Callose_Initial_Big_processed_Big.body.mask.image)
        Set(Callose_ROI_BIG_refined = image)
        Mask2Stencil(Callose_ROI_BIG_refined)
        set(restrict_stencil = stencil)
        Stencil2Objects(restrict_stencil)
        // Find object centres for the big callose
        ObjectsFromCenters(Centers=Callose_Centres.center_spot, RestrictiveStencil=restrict_stencil, image=Callose_Image, MinimumArea= 5)
        ControlBreakingLines_A(1)
        Set(stencil = objects.body)

        //Refine big callose based on intial big callose objects list
        Stencil2Objects()
        FillObjects()
        CalcArea(AutoRecalc=yes, objects=objects)
        ObjectFilter(area > 5)
        CalcIntensity(Image=Callose_Image, Total=no, objects=objects)
        Set(Intensity_Threshold = Callose_Image.mean)
        ObjectFilter(Intensity > Intensity_Threshold * 2.5) // Local value from callose_value objects list
        object_contrast_general(reference = Callose_Image, ContrastDef = "WithoutCyto")
        ObjectFilter(contrast > 0.215 || intensity > Intensity_Threshold * 4.25)
        ObjectFilter(contrast > 0.325 || intensity > Intensity_Threshold * 2.95) // either very bright or have dim surrounding areas
        CalcWidthLength(Upto100=yes)
        Set(Callose_Big_refined = objects)
}

proc EdgeDetection_Small(
image Callose_Image in "the input callose image",
objectlist Callose_Centres in "Input objects list for Callose centers.",
```

```
objectlist Callose_objects in "Input objects list for Callose candidates.",
objectlist ROI_Refined in "Input objects list which defines the ROI.",
objectlist Callose_Small_refined out "Output objects list with refined edge detection."
) //Robatzek Procedures "Perform the edge detection using edge tracing and adaptive thresholding."
{
	//*** Finish refining small callose deposits
	If(Callose_Image.stddev > 0.125) // image contains big intensity difference
		ThresholdXX(TuneTH=1.25, Image=Callose_Image)
		Mask(Threshold=Threshold * 1.625, Image=Callose_Image)
	Else()
		ThresholdXX(TuneTH=1.25, Image=Callose_Image)
		Mask(Threshold=Threshold * 1.525, Image=Callose_Image)
	End()
	Mask2Stencil()
	Stencil2Objects()
	set(Brightest_Area_Ini_Small = objects)

	And(Brightest_Area_Ini_Small.body.mask.image, image = ROI_Refined.body.mask.image)
	// Find small callose ROI
	And(Callose_objects.body.mask.image, image = image)
	Set(Callose_ROI_Small = image)
	Mask2Stencil(Callose_ROI_Small)
	set(restrict_stencil = stencil)

	Stencil2Objects(restrict_stencil)
	CalcArea(AutoRecalc=yes, objects=objects)
	object_contrast_general(reference = Callose_Image, ContrastDef = "WithoutCyto")
	CalcIntensity(Image=Callose_Image, Total= no)
	Set(Intensity_Threshold = objects.intensity.mean) // Use local thresholding
	ObjectFilter(contrast > 0.125 || intensity > Intensity_Threshold * 0.25)
	Set(Callose_Initial_Small_processed = objects)

	ObjectFilter(area > 125, objects = Callose_Initial_Small_processed)
	Set(Callose_Initial_Small_processed_Big_INI = objects)
	ObjectFilter(area >= 25 && area <= 125, objects = Callose_Initial_Small_processed)
	Set(Callose_Initial_Small_processed_Middle_INI = objects)
	ObjectFilter(area < 25, objects = Callose_Initial_Small_processed)
	Set(Callose_Initial_Small_processed_Small_INI = objects)

	// Start to process small objects in small callose deposits
	// Get rid of furry edges
	Set(stencil = Callose_Initial_Small_processed_Small_INI.body)
	CalcErosion(-1, objects = Callose_Initial_Small_processed_Small_INI, Stencil = stencil) // increasing 1 pixel
	RenameAttr(body = stencil_eroded)
	Set(stencil = objects.body)
	CalcErosion(1, objects = objects, Stencil = stencil) // decreasing 1 pixel
	RenameAttr(body = stencil_eroded)
	Mask2Stencil(objects.body.mask.image)
	Stencil2Objects()
```

```
CalcArea()
CalcIntensity(Image=Callose_Image, Total = yes)
CalcAttr(AVG_Intensity, (intensity/area)) // Apply local thresholding
Set(Intensity_Threshold = Callose_Image.mean)
ObjectFilter(AVG_Intensity > Intensity_Threshold * 1.5 && area > 2.5)
// get as many callose deposits as possible
Set(Callose_Initial_Small_processed_Small = objects)

// Start to process big objects in the big callose deposits
// Get rid of furry edges
If(Callose_Image.stddev > 0.125) // image contains big intensity difference
        ThresholdXX(TuneTH=1.25, Image=Callose_Image)
        Mask(Threshold=Threshold * 2.25, Image=Callose_Image)
Else()
        ThresholdXX(TuneTH=1.25, Image=Callose_Image)
        Mask(Threshold=Threshold * 2.15, Image=Callose_Image)
End()

Mask2Stencil()
Stencil2Objects()
// Refine the edge detection of relatively big callose
And(Objects.body.mask.image, image = Callose_Initial_Small_processed_Middle_INI.body.mask.image)

Mask2Stencil(image)
Stencil2Objects()
CalcErosion(-1, objects = objects, Stencil = stencil) // increasing 1 pixel
RenameAttr(body = stencil_eroded)
Set(stencil = objects.body)
CalcErosion(1, objects = objects, Stencil = stencil) // decreasing 1 pixel
RenameAttr(body = stencil_eroded)
Mask2Stencil(objects.body.mask.image)
Stencil2Objects()
CalcArea()
CalcIntensity(Image=Callose_Image, Total= yes)
CalcAttr(AVG_Intensity, (intensity/area)) // Apply local thresholding
Set(Intensity_Threshold = Callose_Image.mean)
ObjectFilter(AVG_Intensity > Intensity_Threshold * 1.5 && area > 2.5)
// get as many callose deposits as possible
Set(Callose_Initial_Small_processed_Middle = objects)

// Start to process big objects in the big callose deposits
// Get rid of furry edges
If(Callose_Image.stddev > 0.125) // image contains big intensity difference
        ThresholdXX(TuneTH=1.25, Image=Callose_Image)
        Mask(Threshold=Threshold * 3.05, Image=Callose_Image)
Else()
        ThresholdXX(TuneTH=1.25, Image=Callose_Image)
        Mask(Threshold=Threshold * 2.95, Image=Callose_Image)
End()
```

```
Mask2Stencil()
Stencil2Objects()
// Refine the edge detection of relatively big callose
And(Objects.body.mask.image, image = Callose_Initial_Small_processed_Big_INI.body.mask.image)

Mask2Stencil(image)
Stencil2Objects()
CalcErosion(-2, objects = objects, Stencil = stencil) // increasing 1 pixel
RenameAttr(body = stencil_eroded)
Mask2Stencil(objects.body.mask.image)
Stencil2Objects()
CalcErosion(2, objects = objects, Stencil = stencil) // decreasing 1 pixel
RenameAttr(body = stencil_eroded)
Mask2Stencil(objects.body.mask.image)
Stencil2Objects()
CalcArea()
CalcIntensity(Image=Callose_Image, Total= yes)
CalcAttr(AVG_Intensity, (intensity/area)) // Apply local thresholding
Set(Intensity_Threshold = Callose_Image.mean)
ObjectFilter(AVG_Intensity > Intensity_Threshold * 1.75)
// get as many callose deposits as possible
Set(Callose_Initial_Small_processed_Big = objects)

// Rebuild the callose objects list based on the refined callose centres
Or(Callose_Initial_Small_processed_Small.body.mask.image, image = Callose_Initial_Small_processed_Middle.body.mask.image)
Or(image, image = Callose_Initial_Small_processed_Big.body.mask.image)
Set(Callose_ROI_SMALL = image)
Mask2Stencil(Callose_ROI_SMALL)
set(restrict_stencil = stencil)
Stencil2Objects(restrict_stencil)
CalcWidthLength(Upto100=yes)

// Find object centres for the small callose
ObjectsFromCenters(Centers=Callose_Centres.center_spot, RestrictiveStencil=restrict_stencil, image=Callose_Image, MinimumArea= 5)
ControlBreakingLines_A(1)
Set(stencil = objects.body)

//Start to refine small callose
Set(stencil = objects.body)
Stencil2Objects()
CalcIntensity(Image=Callose_Image, Total=no, objects=objects)
Set(Intensity_Threshold = Callose_Image.mean)
ObjectFilter(Intensity > Intensity_Threshold * 1.75) // Local value from callose_value objects list
CalcArea()
ObjectFilter(area >= 5)

calcArea(border, AutoRecalc=yes, objects=objects)
object_contrast_general(reference = Callose_Image, ContrastDef = "WithoutCyto")
```

```
        ObjectFilter((area < 32.5 && (contrast > 0.125 || intensity > Intensity_Threshold * 2.25)) || (area >= 32.5 && (contrast > 0.15 || intensity > Intensity_Threshold * 2.5)))
        // either very bright or have dim surrounding areas

        // The small callose objects have been expanded
        CalcAttr(Roundness, 4 * pi * area / (border_area * border_area) * 100)
        ObjectFilter(Roundness > 90 || contrast > 0.195 || intensity > Intensity_Threshold * 2.25)
        ObjectFilter(Roundness > 115 || ((intensity > Intensity_Threshold * 2.25 || contrast > 0.325) && area >= 25) || area < 25)
        // normally roundness should be over 90

        CalcWidthLength(Upto100=yes)
        ObjectFilter(half_width > 1 && full_length > 1.5)
        ObjectFilter((full_length > 3.5 && full_length < 13.75 && half_width > 1.5) || (contrast > 0.175 || intensity > Intensity_Threshold * 2.75))

        CalcAttr(WL_Ratio, full_length/(half_width * 2))
        ObjectFilter(!(WL_Ratio >= 3 && contrast < 0.35 && area < 75)) // exceptional cases
        DeleteAttr("outerzone", objects = objects)
        DeleteAttr("zone", objects = objects)
        DeleteAttr("top_spot1", objects = objects)
        DeleteAttr("top_spot2", objects = objects)
        Set(Callose_Small_refined = objects)
}//*** Internal functions complete***//
```
