# Supplementary material for: CalloseMeasurer: a novel software solution to measure callose deposition and recognise spreading callose patterns
Source: Plant Methods. 2012 Dec 17;8:49. doi: 10.1186/1746-4811-8-49 (PMC3571893; doi:10.1186/1746-4811-8-49)
Supplement: Additional file 3 — Examples of CSV files generated for results. [file 1746-4811-8-49-S3.pdf]

# 1 EXAMPLES OF CSV FILES GENERATED FOR OVERALL RESULTS

**Table S1.** An overall CSV file with the quantification of spreading callose networks

| Image_Name | Callose_Number | Average_Callose_Size | Average_Callose_Intensity | Calculated_Spreading_Length | Total_Callose_area |
|---|---|---|---|---|---|
| image18 | 186 | 32.3172 | 87.98812 | 72 | 6011 |
| image19 | 408 | 30.84314 | 96.8975 | 1018.635 | 12584 |
| image5 | 130 | 40.63846 | 84.47546 | 57 | 5283 |
| image53 | 359 | 32.50975 | 99.86398 | 414.4041 | 11671 |
| image58_ctl | 382 | 41.08115 | 125.6333 | 1009.528 | 15693 |

**Table S2.** An overall CSV file without the quantification of spreading callose networks

| Image_Name | Callose_Number | Average_Callose_Size | Average_Callose_Intensity |
|---|---|---|---|
| image18 | 186 | 32.3172 | 87.98812 |
| image19 | 408 | 30.84314 | 96.8975 |
| image5 | 130 | 40.63846 | 84.47546 |
| image53 | 359 | 32.50975 | 99.86398 |
| image58_ctl | 382 | 41.08115 | 125.6333 |

# 2 AN EXAMPLE OF CSV FILES GENERATED FOR EVERY PROCESSED IMAGE

**Table S3.** A CSV file which contains the quantification of every callose deposit

| Image_Name | Callose_Index | Size | Circularity | Intensity |
|---|---|---|---|---|
| image5 | 1 | 99 | 1.294558 | 132.2828 |
| image5 | 2 | 13 | 2.552544 | 36.30769 |
| image5 | 3 | 17 | 2.136283 | 41.47059 |
| image5 | 4 | 13 | 2.552544 | 43.30769 |
| image5 | 5 | 61 | 1.738205 | 66.72131 |
| image5 | 6 | 19 | 1.658063 | 42.57895 |
| image5 | 7 | 28 | 1.217503 | 47.96429 |
| image5 | 8 | 37 | 1.608843 | 55.10811 |
| image5 | 9 | 20 | 1.745329 | 43.65 |
| image5 | 10 | 80 | 1.608495 | 140.5625 |
| image5 | 11 | 106 | 1.386093 | 198.5189 |
| image5 | 12 | 24 | 1.784573 | 44.83333 |
| image5 | 13 | 38 | 1.322776 | 85.26316 |
| image5 | 14 | 36 | 1.396263 | 86.05556 |
| image5 | 15 | 26 | 1.130539 | 122.5 |
| image5 | 16 | 16 | 2.010619 | 39.9375 |
| image5 | 17 | 61 | 1.583778 | 88.5082 |
| image5 | 18 | 56 | 1.759292 | 102.5714 |

| | | | | |
|---|---|---|---|---|
| image5 | 19 | 27 | 1.325359 | 74.85185 |
| image5 | 20 | 41 | 1.590189 | 47.41463 |
| image5 | 21 | 13 | 2.552544 | 115.6154 |
| image5 | 22 | 46 | 1.601255 | 90 |
| image5 | 23 | 14 | 2.171965 | 40.42857 |
| image5 | 24 | 157 | 1.44114 | 217.4395 |
| image5 | 25 | 100 | 1.602853 | 146.36 |
| image5 | 26 | 29 | 1.619666 | 54.03448 |
| image5 | 27 | 31 | 1.34795 | 59.41935 |
| image5 | 28 | 58 | 1.505887 | 71.94828 |
| image5 | 29 | 28 | 1.563815 | 123.4286 |
| image5 | 30 | 36 | 1.130973 | 84.47222 |
| image5 | 31 | 46 | 1.310778 | 79.82609 |
| image5 | 32 | 13 | 2.552544 | 68.76923 |
| image5 | 33 | 124 | 1.272024 | 178.9839 |
| image5 | 34 | 22 | 1.919862 | 58.09091 |
| image5 | 35 | 25 | 1.858931 | 41.68 |
| image5 | 36 | 19 | 1.973232 | 61.21053 |
| image5 | 37 | 13 | 2.552544 | 39.76923 |
| image5 | 38 | 37 | 1.435048 | 44.75676 |
| image5 | 39 | 30 | 1.675516 | 52.6 |
| image5 | 40 | 165 | 1.233463 | 166.5576 |
| image5 | 41 | 31 | 1.34795 | 42.70968 |
| image5 | 42 | 13 | 2.016825 | 126 |
| image5 | 43 | 17 | 1.765523 | 40.11765 |
| image5 | 44 | 35 | 1.718058 | 71.94286 |
| image5 | 45 | 27 | 1.731082 | 45.07407 |
| image5 | 46 | 202 | 1.057229 | 177.4554 |
| image5 | 47 | 19 | 1.973232 | 42.05263 |
| image5 | 48 | 33 | 1.619884 | 59.36364 |
| image5 | 49 | 32 | 1.787217 | 44.46875 |
| image5 | 50 | 38 | 1.322776 | 42.73684 |
| image5 | 51 | 30 | 1.923424 | 87.5 |
| image5 | 52 | 37 | 1.608843 | 103.027 |
| image5 | 53 | 16 | 1.661669 | 71.25 |
| image5 | 54 | 47 | 1.116483 | 82.06383 |
| image5 | 55 | 46 | 1.445133 | 84.47826 |
| image5 | 56 | 24 | 1.784573 | 81.70833 |
| image5 | 57 | 20 | 1.745329 | 153.3 |
| image5 | 58 | 66 | 1.226894 | 206.8485 |
| image5 | 59 | 53 | 1.259013 | 206.6415 |
| image5 | 60 | 11 | 2.159845 | 113.4545 |
| image5 | 61 | 34 | 1.478397 | 165.2353 |
| image5 | 62 | 19 | 1.658063 | 40.15789 |
| image5 | 63 | 14 | 2.171965 | 76.28571 |
| image5 | 64 | 27 | 1.507964 | 99.92593 |

| | | | | |
|---|---|---|---|---|
| image5 | 65 | 68 | 1.48353 | 97.57353 |
| image5 | 66 | 19 | 1.973232 | 83.94737 |
| image5 | 67 | 29 | 1.619666 | 94.72414 |
| image5 | 68 | 62 | 1.472807 | 83.66129 |
| image5 | 69 | 147 | 1.098903 | 158.6122 |
| image5 | 70 | 22 | 1.919862 | 79.72727 |
| image5 | 71 | 26 | 1.452114 | 87.69231 |
| image5 | 72 | 23 | 1.284562 | 80.82609 |
| image5 | 73 | 37 | 1.287966 | 42.62162 |
| image5 | 74 | 26 | 1.933288 | 60.53846 |
| image5 | 75 | 36 | 1.565361 | 93.86111 |
| image5 | 76 | 23 | 1.710216 | 50.30435 |
| image5 | 77 | 24 | 1.784573 | 80.54167 |
| image5 | 78 | 15 | 1.884956 | 76.13333 |
| image5 | 79 | 37 | 1.435048 | 105.7297 |
| image5 | 80 | 15 | 1.884956 | 74.2 |
| image5 | 81 | 20 | 1.487144 | 74.55 |
| image5 | 82 | 22 | 1.635859 | 76.31818 |
| image5 | 83 | 12 | 1.861685 | 75.75 |
| image5 | 84 | 38 | 1.652326 | 133.2105 |
| image5 | 85 | 36 | 1.565361 | 48.38889 |
| image5 | 86 | 21 | 1.561502 | 86.09524 |
| image5 | 87 | 31 | 1.987538 | 72.41935 |
| image5 | 88 | 31 | 1.731367 | 111.5484 |
| image5 | 89 | 66 | 1.713596 | 137.2424 |
| image5 | 90 | 13 | 2.552544 | 110.2308 |
| image5 | 91 | 30 | 1.472622 | 102.9667 |
| image5 | 92 | 35 | 1.718058 | 116.7143 |
| image5 | 93 | 74 | 1.61443 | 221.8378 |
| image5 | 94 | 44 | 1.706544 | 87.02273 |
| image5 | 95 | 16 | 1.661669 | 73.5 |
| image5 | 96 | 42 | 1.628974 | 93.04762 |
| image5 | 97 | 47 | 1.822899 | 146.383 |
| image5 | 98 | 13 | 2.552544 | 110.3846 |
| image5 | 99 | 64 | 1.661669 | 79.64063 |
| image5 | 100 | 53 | 1.665044 | 115.0566 |
| image5 | 101 | 41 | 1.782772 | 77.46341 |
| image5 | 102 | 62 | 1.609742 | 107.9355 |
| image5 | 103 | 90 | 1.256637 | 92.18889 |
| image5 | 104 | 29 | 1.619666 | 46.37931 |
| image5 | 105 | 18 | 1.869377 | 74.55556 |
| image5 | 106 | 76 | 1.658063 | 77.02632 |
| image5 | 107 | 14 | 2.171965 | 71 |
| image5 | 108 | 28 | 1.374447 | 40.64286 |
| image5 | 109 | 26 | 1.452114 | 40.84615 |
| image5 | 110 | 26 | 1.452114 | 44.96154 |

| | | | | |
|---|---|---|---|---|
| image5 | 111 | 27 | 1.731082 | 64.51852 |
| image5 | 112 | 33 | 1.619884 | 43.93939 |
| image5 | 113 | 47 | 1.636065 | 66.3617 |
| image5 | 114 | 25 | 1.858931 | 76.72 |
| image5 | 115 | 13 | 2.552544 | 118.7692 |
| image5 | 116 | 24 | 1.538739 | 75.95833 |
| image5 | 117 | 18 | 1.570796 | 40.94444 |
| image5 | 118 | 36 | 1.396263 | 47.77778 |
| image5 | 119 | 39 | 1.111312 | 41.20513 |
| image5 | 120 | 53 | 1.665044 | 63.20755 |
| image5 | 121 | 24 | 1.784573 | 40.95833 |
| image5 | 122 | 33 | 1.619884 | 61.18182 |
| image5 | 123 | 37 | 1.435048 | 40.48649 |
| image5 | 124 | 31 | 1.521709 | 42.32258 |
| image5 | 125 | 262 | 1.428988 | 230.9351 |
| image5 | 126 | 13 | 2.552544 | 37.07692 |
| image5 | 127 | 25 | 1.602853 | 42.88 |
| image5 | 128 | 47 | 1.822899 | 70.55319 |
| image5 | 129 | 16 | 2.010619 | 39.125 |
| image5 | 130 | 38 | 1.473834 | 41.28947 |
