# Supplementary material for: CalloseMeasurer: a novel software solution to measure callose deposition and recognise spreading callose patterns
Source: Plant Methods. 2012 Dec 17;8:49. doi: 10.1186/1746-4811-8-49 (PMC3571893; doi:10.1186/1746-4811-8-49)
Supplement: Additional file 4 — CalloseMeasurer analysis results for batch processing images and for constructing spreading callose networks. [file 1746-4811-8-49-S4.pdf]

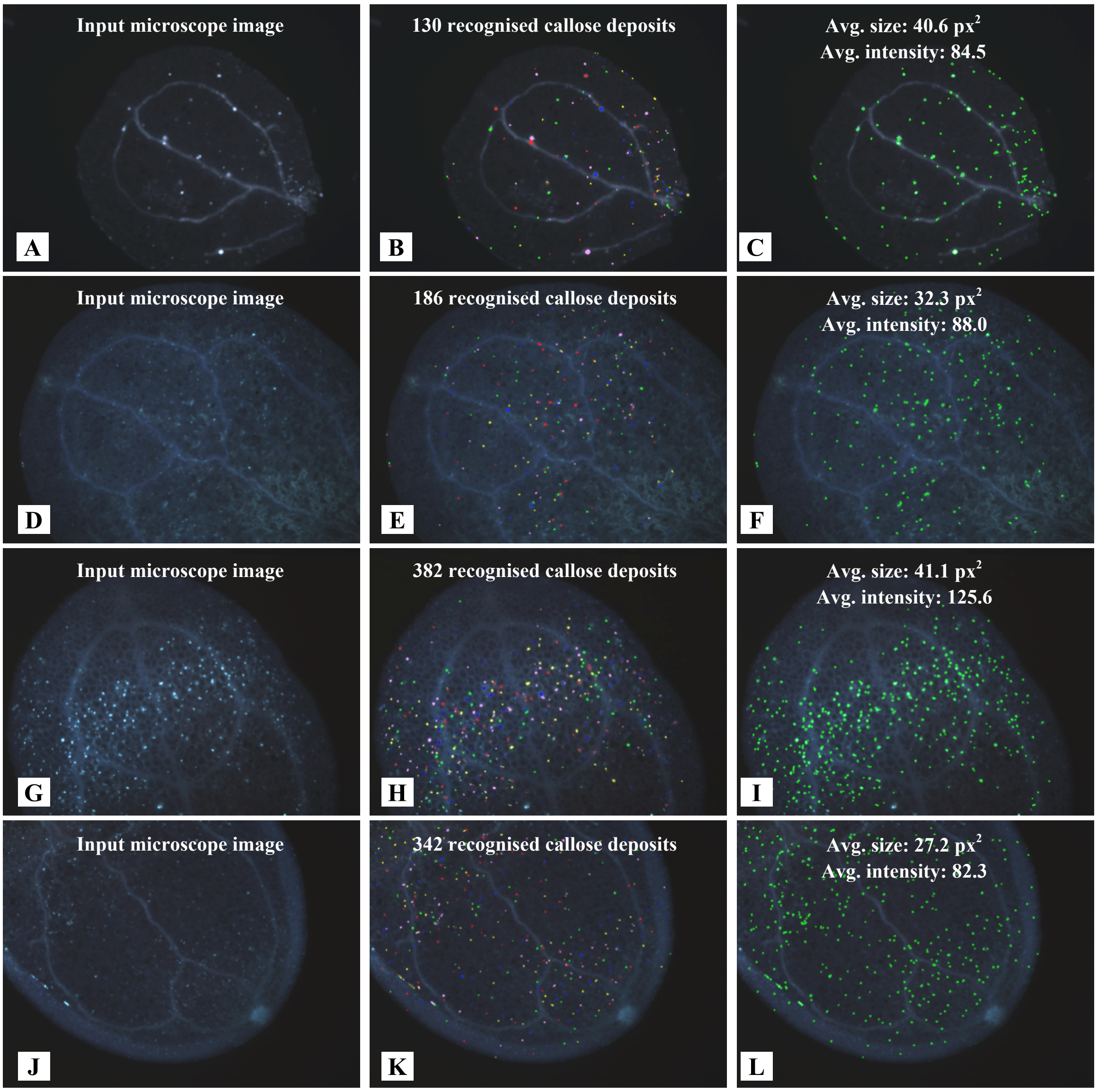

Fig A1. CalloseMeasurer analysis results for batch processing images from flg22-induced callose.

(A, D, G, J) The algorithm reads a series of image files and splits them into three planes – hue, saturation, and intensity value. Only intensity planes are used in the analysis.

(B, E, H, K) The algorithm detects callose deposits, which are randomly colored.

(C, F, I, L) Quantifiable results (e.g., size, shape, and intensity) are exported to a CSV file, together with a set of processed images (in PNG format) with callose deposits highlighted with green.

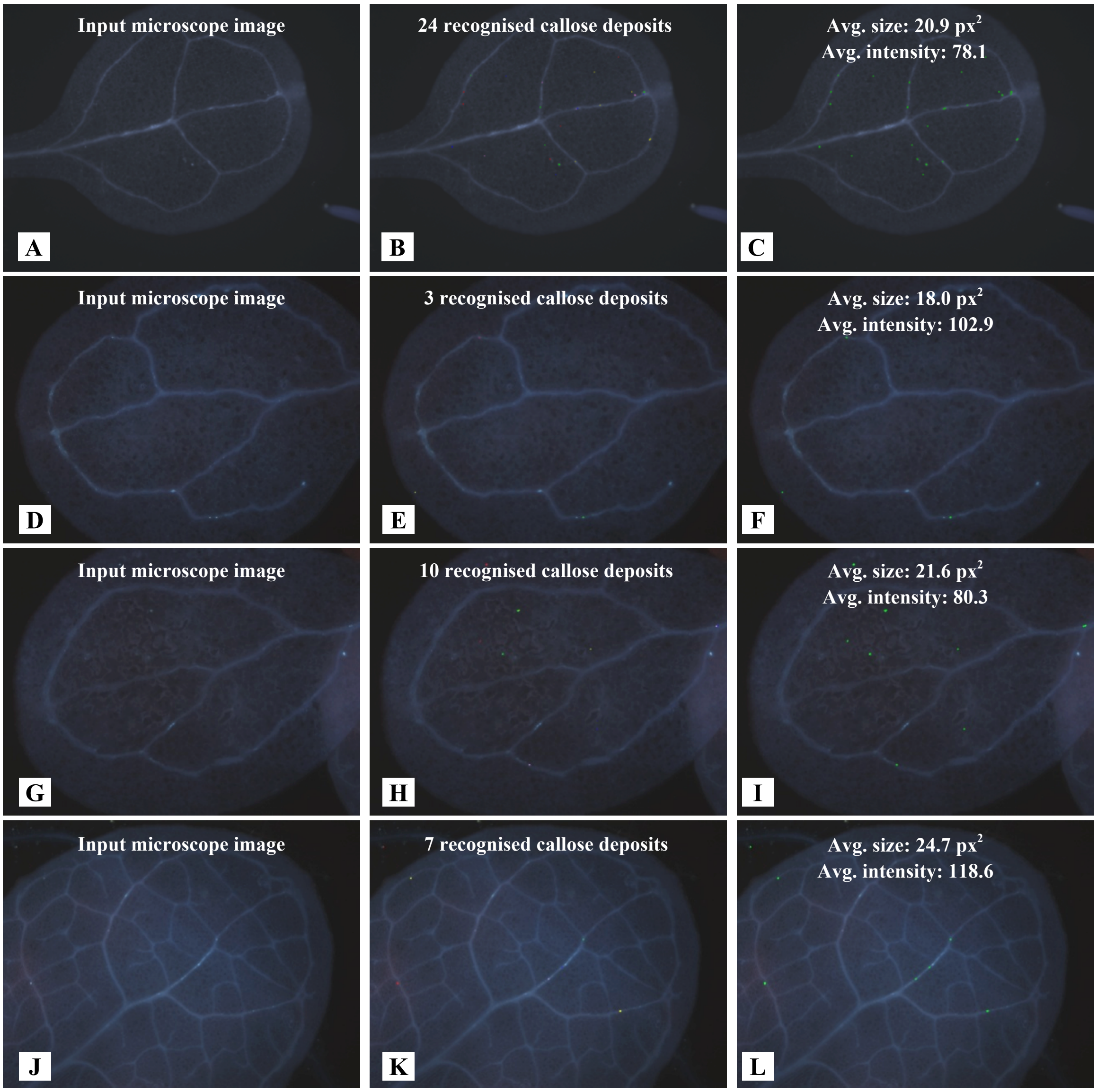

Fig. A2. CalloseMeasurer analysis results for batch processing images from control treatments.

(A, D, G, J) The algorithm reads a series of image files and splits them into three planes – hue, saturation, and intensity value. Only intensity planes are used in the analysis.

(B, E, H, K) The algorithm detects callose deposits, which are randomly colored.

(C, F, I, L) Quantifiable results (e.g., size, shape, and intensity) are exported to a CSV file, together with a set of processed images (in PNG format) with callose deposits highlighted with green.

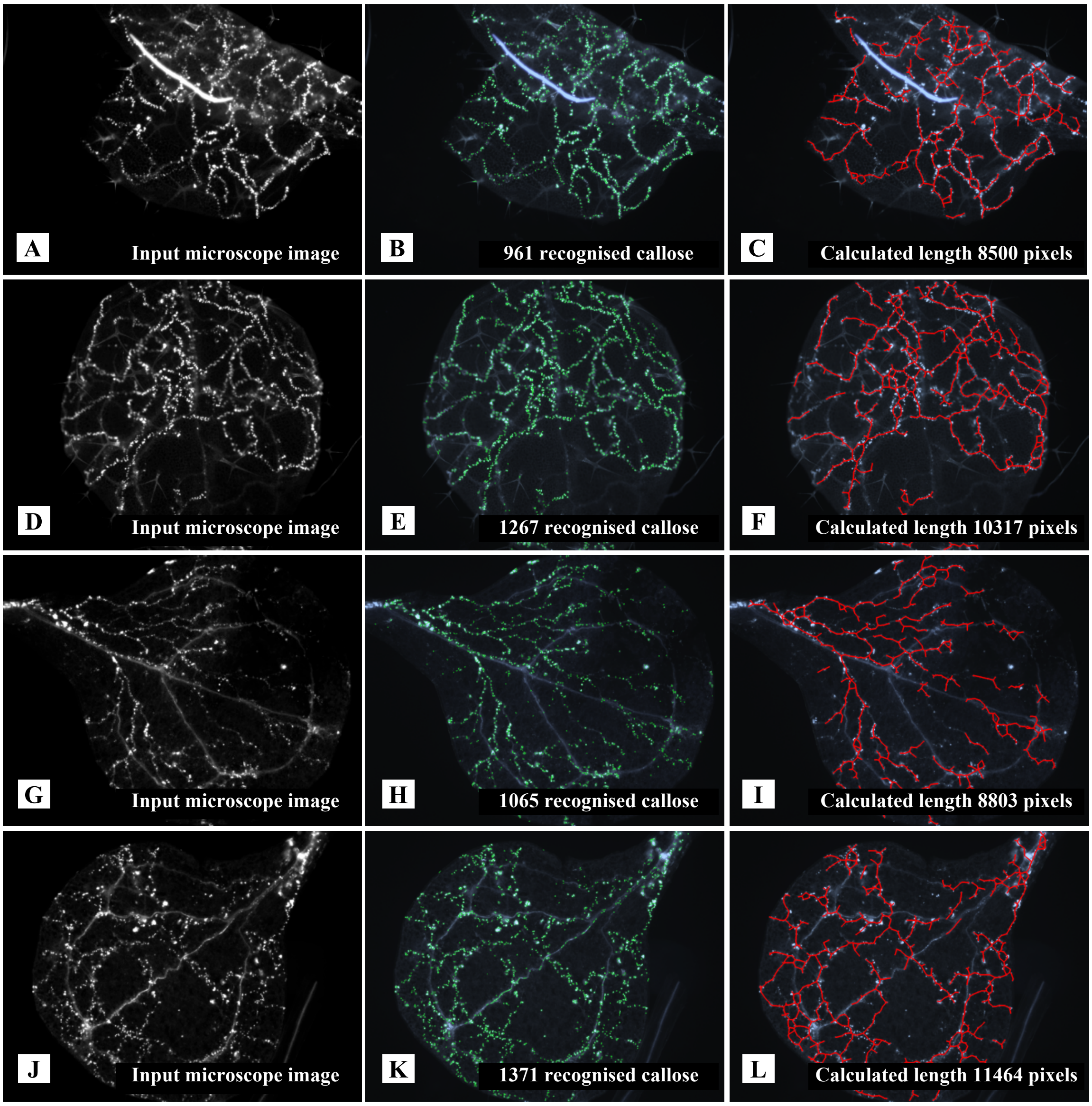

Fig. S3. CalloseMeasurer analysis results for constructing spreading callose networks

(A, D, G, J) The algorithm reads a series of image files and splits them into three planes – hue, saturation, and intensity value. Only intensity planes are used in the analysis.

(B, E, H, K) The algorithm detects callose deposits, which are randomly colored.

(C, F, I, L) Spreading callose networks are constructed based on recognized callose deposits. Features such as length and perimeter are exported to a CSV file, together with a set of images (in PNG format) highlighted with pathogen growth patterns (colored red).
